# CoSe_2_ Nanoparticles Encapsulated by N‐Doped Carbon Framework Intertwined with Carbon Nanotubes: High‐Performance Dual‐Role Anode Materials for Both Li‐ and Na‐Ion Batteries

**DOI:** 10.1002/advs.201800763

**Published:** 2018-10-17

**Authors:** Jun Yang, Hongcheng Gao, Shuang Men, Zhenqing Shi, Zhang Lin, Xiongwu Kang, Shaowei Chen

**Affiliations:** ^1^ Guangzhou Key Laboratory for Surface Chemistry of Energy Materials New Energy Research Institute School of Environment and Energy South China University of Technology Guangzhou 510006 China; ^2^ Guangdong Engineering and Technology Research Center for Environmental Nanomaterials School of Environment and Energy South China University of Technology Guangzhou Guangdong 510006 China; ^3^ Department of Chemistry and Biochemistry University of California 1156 High Street Santa Cruz CA 95064 USA

**Keywords:** carbonate electrolytes, charge/discharge mechanisms, CoSe_2_, dual‐role anode materials, Li‐ion batteries, sodium‐ion batteries

## Abstract

It is of fundamental and technological significance to develop dual‐role anode materials for both lithium‐ion batteries (LIBs) and sodium‐ion batteries (SIBs) with high performance. Here, a composite material based on CoSe_2_ nanoparticles encapsulated in N‐doped carbon framework intertwined with carbon nanotubes (CoSe_2_@N‐CF/CNTs) is prepared successfully from cobalt‐based zeolitic imidazolate framework (ZIF‐67). As anode materials for LIBs, CoSe_2_@N‐CF/CNTs composites deliver a reversible capacity of 428 mAh g^−1^ even after 500 cycles at a current density of 1 A g^−1^ with almost 100% Coulombic efficiency. The charge and discharge mechanisms of CoSe_2_ are characterized using ex situ X‐ray diffraction and Raman analysis, from which the lithiation products of CoSe_2_ are found to be Li*_x_*CoSe_2_ and Li_2_Se, which are further converted to CoSe_2_ upon delithiation. The CoSe_2_@N‐CF/CNTs composites also demonstrate excellent electrochemical performance as anode materials for SIBs with a carbonate‐based electrolyte, with specific capacities of 606 and 501 mAh g^−1^ at 0.1 and 1 A g^−1^ in the 100th cycle. The electrochemical performance of the anode materials is further studied by pseudocapacitance and galvanostatic intermittent titration technique (GITT) measurements. This work may be exploited for the rational design and development of dual‐role anode materials for both Li‐ and Na‐ion batteries.

## 1 Introduction

Recently, sodium‐ion batteries (SIBs) have been attracting significant interests of the scientific communities, because SIBs are believed to be a viable alternative for lithium‐ion batteries (LIBs), especially in the field of large‐scale energy storage.^1^ Notably, the redox potential of sodium is only 0.3 V higher than that of lithium, and sodium is of high natural abundance and low costs. However, as the ionic radius of Na is larger than that of Li (0.102 vs 0.076 nm), the electrode host materials that are good for LIBs are not suitable for SIBs.[[qv: 1c,2]] Yet, it is generally believed that the electrode materials for LIBs and SIBs are relevant to each other and it is highly desired to design electrode host materials that may be used for both LIBs and SIBs.[[qv: 1d,3]]

Composite nanomaterials based on transition‐metal selenides encapsulated in carbonaceous materials, especially those derived from metal–organic frameworks (MOFs), have demonstrated great promise as dual‐role anode materials for both LIBs and SIBs,^4^ primarily due to the high electric conductivity and theoretical capacity.^5^ With the unique architecture of the carbon matrix, not only can the transport kinetics of ions and electrons be accelerated, but also the structural collapse resulting from volume changes can be significantly alleviated, thus leading to enhanced insertion/extraction reversibility and improved Li^+^/Na^+^ storage capacity.[[qv: 3b,6]] For example, MoSe_2_ nanosheets supported by porous hollow carbon spheres have been prepared by a three‐step method and delivered a capacity of 681 and 580 mAh g^−1^ for LIBs and SIBs, respectively, after 100 cycles.^7^ CoSe@porous carbon polyhedra (PCP) composites, prepared through the carbonization treatment of cobalt‐based zeolitic imidazolate framework (ZIF‐67) and further selenization at 650 °C in a N_2_ atmosphere, delivered a capacity of 675 mAh g^−1^ after 100 cycles at a current rate of 0.2 A g^−1^ for LIBs and 341 mAh g^−1^ after 100 cycles at 0.1 A g^−1^ for SIBs.[[qv: 3b]]

CoSe_2_/C composites have been prepared in different conditions and demonstrated excellent electrochemical performance as anode materials for SIBs. For example, the application of a H_2_/Ar atmosphere during carbonization of ZIF‐67 at 550 °C resulted in the formation of a N‐doped carbon framework intertwined with carbon nanotubes.[[qv: 2b]] Both orthorhombic and cubic CoSe_2_ were observed in X‐ray diffraction (XRD) measurements. As anode materials for Na‐storage, they displayed a capacity of 424 mAh g^−1^ after 100 cycles at a current rate of 0.1 A g^−1^ and 300.2 mAh g^−1^ after 400 cycles at 1 A g^−1^. In another study, Qiu and co‐workers^8^ carbonized ZIF‐67 in H_2_/Ar at 600 °C to form a carbon matrix embedded with Co nanoparticles bridged by carbon nanotubes (Co@C/CNTs). Vaporizing Se powder at 360 °C and further reaction of Se vapor with Co nanoparticles imbedded inside the carbon matrix for 10 h resulted in the formation of CoSe_2_@C/CNTs composite. When served as the anodes for SIBs, CoSe_2_@C/CNTs exhibited a stable cycling performance with a reversible capacity of 390 mAh g^−1^ over 1000 cycles at 1 A g^−1^. Although such CoSe_2_/C composites demonstrated excellent cycling stability as anode materials for SIBs, the gas–solid flow reaction requires continuous input of high energy and is not suitable for large‐scale industrial production. Most importantly, the investigation of CoSe_2_ as dual anode materials for both LIBs and SIBs, their charge and discharge mechanisms, as well as the electrolyte effect on the battery performance have remained largely unexplored.

Inspired by the abovementioned structural modification strategies, in this work, CoSe_2_ nanoparticles encapsulated into a N‐doped carbon framework that was intertwined with carbon nanotubes (CoSe_2_@N‐CF/CNTs) was synthesized successfully by a facile MOF‐engaged approach, which is suitable for mass production in industry. The derived CoSe_2_@N‐CF/CNTs composite materials demonstrated superior electrochemical performance as anode materials for both LIBs and SIBs.

## 2 Results and Discussion

### 2.1 Structural Analysis

**Figure 1:**
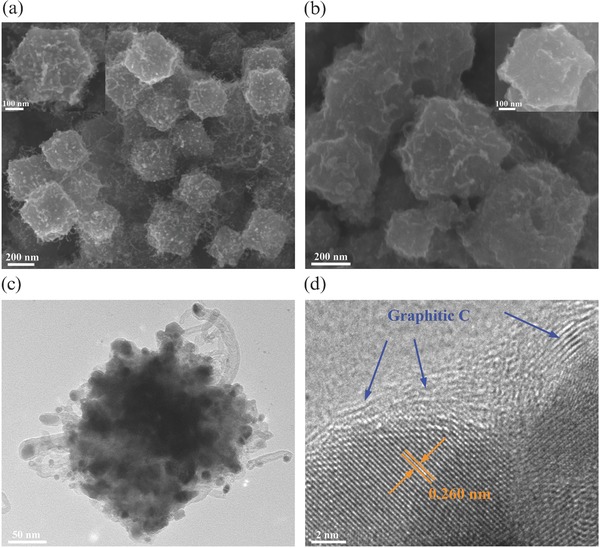
SEM images of a) Co@N‐CF/CNTs and b) CoSe_2_@N‐CF/CNTs. c,d) TEM images of CoSe_2_@N‐CF/CNTs.

The scanning electron microscopy (SEM) images of the prepared polyhedral ZIF‐67 template were shown in Figure S1 (Supporting Information), which showed a smooth surface. From **Figure** **1**a, after pyrolysis under a reductive Ar/H_2_ atmosphere at 700 °C for 3 h, the ZIF‐67 precursors were converted into carbon polyhedra intertwined with a large number of surficial CNTs (Co@N‐CF/CNTs). The growth of interconnected CNTs on the external surface of the carbon framework was attributed to the catalytic effect of Co nanoparticles under the reducing gas streams,[[qv: 2b,8,9]] which not only provided an interlaced conductive network to facilitate electrolyte penetration and charge transportation, but also effectively mitigated the volume expansion and maintained the structural integrity of the carbon matrix.[[qv: 2b,8,9]] Figure 1b,c shows the SEM and transmission electron microscopy (TEM) images of the CoSe_2_@N‐CF/CNTs composite, which mostly reserved the original structural morphology of Co@N‐CF/CNTs upon the selenization at 300 °C. The energy‐dispersive X‐ray spectra (EDX) of CoSe_2_@N‐CF/CNTs were shown in Figure S2 (Supporting Information). The uniform distribution of C, O, N, Co, and Se elements can be readily observed, and the atomic ratio of Se/Co (2.04) strongly supported the formation of CoSe_2_, which was well wrapped by the N‐doped carbonaceous matrix. The encapsulation of CoSe_2_ nanoparticles by carbon layers was further evidenced in high‐resolution TEM (HRTEM) measurements (Figure 1d). The interplanar spacing of 0.260 nm was in agreement with that of the (111) crystal plane of orthorhombic CoSe_2_.

**Figure 2:**
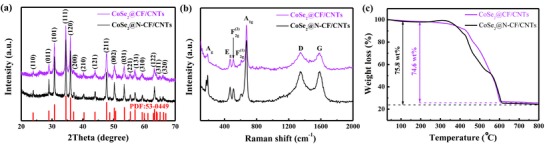
a) XRD patterns, b) Raman spectra, and c) TGA curves of CoSe_2_@N‐CF/CNTs and CoSe_2_@CF/CNTs.

The successful synthesis of ZIF‐67 crystals is also confirmed in XRD measurements (Figure S3a, Supporting Information).^10^ Figure S3b (Supporting Information) shows the diffraction peaks of Co@N‐CF/CNTs and Co@CF/CNTs derived from carbonization treatment of ZIF‐67 at different temperatures, where the diffraction peaks for ZIF‐67 crystal vanished completely, and three characteristic peaks for the (111), (200), and (220) lattice facets of metallic cobalt at 2θ = 44.22°, 51.52°, and 75.85° emerged, suggesting the formation of cobalt nanoparticles.^11^ These diffraction peaks of Co nanoparticles became increasingly intensified and sharpened with increasing calcination temperature, indicating enhanced crystallinity at elevated temperatures.[[qv: 5d]] According to the Scherrer's equation, *d* = 0.89λ/βcosθ, where *d* presents the average size of the crystallites, λ is the X‐ray wavelength, β is the full width at half‐maximum (FWHM) for the Co(111) peak located at the diffraction angle θ, the average crystallite sizes were calculated to be 8.78 and 17.89 nm for Co@N‐CF/CNTs and Co@CF/CNTs, respectively. The increment of calcination temperature resulted in larger sizes of Co crystallite. The tiny diffraction peak at 2θ = 26.38° was associated with the graphitic carbon, owing to the improved graphitic crystallinity during carbonization.[[qv: 11b,12]] **Figure** **2**a shows the XRD patterns of CoSe_2_@N‐CF/CNTs and CoSe_2_@CF/CNTs prepared at an optimized temperature at 300 °C for 12 h, which are well indexed to orthorhombic CoSe_2_ (PDF No. 53‐0449; *Pnnm*(58), *a* = 3.643 Å, *b* = 4.896 Å, *c* = 5.821 Å). Co@N‐CF/CNTs annealed with Se powder at elevated temperatures (400, 500, and 600 °C) displayed the reflections of CoSe at 2θ = 37.76° and 51.91° (PDF No. 09‐0234; *Pa*3(205), *a* = 5.843 Å, *b* = 5.843 Å, *c* = 5.843 Å), as shown in Figure S4a (Supporting Information). Figure 2b displays the Raman spectra of CoSe_2_@N‐CF/CNTs and CoSe_2_@CF/CNTs, where the two peaks at 672 and 188 cm^−1^ are attributed to the A_1g_ and A_g_ modes of CoSe_2_,^8^, ^13^ while the three minor bands at 469, 511, and 607 cm^−1^ are possibly associated with the E_g_, $F_{2g}^{\left(2\right)}$, and $F_{2g}^{\left(1\right)}$ modes of Co_3_O_4_ caused by slight surface oxidation.^8^, ^13^, ^14^ The two peaks at 1346 and 1585 cm^−1^ are due to the characteristic D and G bands of the carbon framework.[[qv: 9a,12,15]] CoSe_2_@N‐CF/CNTs exhibited a somewhat lower *I*_D_/*I*_G_ ratio than CoSe_2_@CF/CNTs (0.98 vs 1.02), implying a higher degree of graphitization, which was favorable for improved electrical conductivity.^16^

The weight percentages of CoSe_2_ in CoSe_2_@N‐CF/CNTs and CoSe_2_@CF/CNTs composites were determined by thermogravimetric analysis (TGA) measurements in an O_2_ atmosphere at a heating rate of 10 °C min^−1^ from 30 to 800 °C, as shown in Figure 2c. We can see that CoSe_2_@N‐CF/CNTs exhibited one step of weight increase and multiple steps of weight loss. The small mass loss below 200 °C was attributed to the evaporation of adsorbed water. The slight weight increase between 200 and 350 °C was likely due to the oxidation of cobalt and selenium species, partially transforming CoSe_2_ into CoSe_2_O_5_.[[qv: 3a,5d,17]] The major mass loss between 350 and 800 °C was ascribed to the oxidation of CoSe_2_ to Co_3_O_4_, the combustion of carbon, and transformation of CoSe_2_O_5_ to Co_3_O_4_.[[qv: 3a,5a,18]] On the basis of the final product Co_3_O_4_ (XRD pattern in Figure S4b in the Supporting Information), the weight percentages of CoSe_2_ in CoSe_2_@N‐CF/CNTs and CoSe_2_@CF/CNTs were calculated to be 65.4 and 68.6 wt%, respectively. In addition, N_2_ adsorption–desorption isotherms of CoSe_2_@N‐CF/CNTs and CoSe_2_@CF/CNTs were shown in Figure S5 (Supporting Information), which are characteristic of type IV isotherms with hysteresis loops at high relative pressures, indicating the formation of a mesoporous configuration.[[qv: 5d,19]] The specific surface areas of Co@N‐CF/CNTs and Co@CF/CNTs derived from the isotherms were 277.59 and 237.96 m^2^ g^−1^, respectively, with the pore size of 2–6 nm. The slightly smaller specific surface area of Co@CF/CNTs than Co@N‐CF/CNTs was likely due to the aggregation of cobalt nanoparticles at higher temperatures.[[qv: 5d,20]] Selenization of Co@N‐CF/CNTs and Co@CF/CNTs remarkably reduced the specific surface area and total pore volume of the composites (Table S1, Supporting Information) due to the formation of CoSe_2_ nanoparticles. The CoSe_2_@N‐CF/CNT sample has the largest average pore diameter of 31.39 nm and the submaximal total pore volume of 0.134 cm^3^ g^−1^, which was favorable to facilitate access of the electrode surface by electrolyte and thus to accelerate ion diffusion, electron transfer, and electrolyte infiltration. Importantly, the porous structure may also effectively buffer the large volume expansion during repeated charge/discharge process, leading to enhanced Li^+^/Na^+^ storage capacity and rate capability.[[qv: 3a,5d,21]]

**Figure 3:**
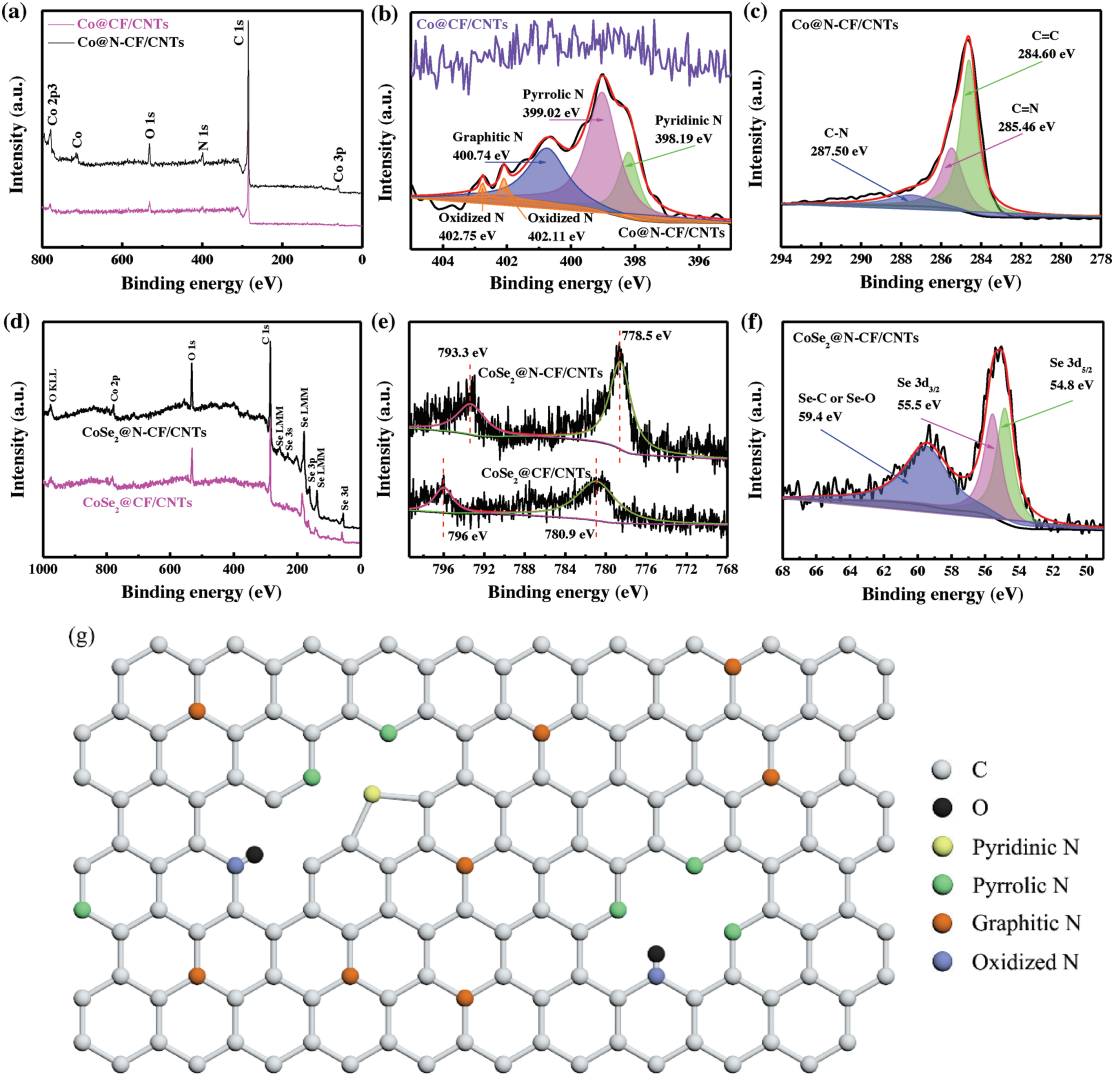
a) Survey XPS and b) high‐resolution N 1s spectra of Co@CF/CNTs (top) and Co@N‐CF/CNTs (bottom). c) High‐resolution C 1s spectrum of Co@N‐CF/CNTs. d) Survey XPS and e) high‐resolution Co 2p spectra of CoSe_2_@N‐CF/CNTs and CoSe_2_@CF/CNTs. f) High‐resolution Se 3d spectrum of CoSe_2_@N‐CF/CNTs. g) Schematic representation of the N configuration in carbon matrix.

The chemical compositions of Co@N‐CF/CNTs and Co@CF/CNTs were then examined by X‐ray photoelectron spectra (XPS) measurements. The full survey spectra in **Figure** **3**a indicate the presence of Co, C, O, and N elements in Co@N‐CF/CNTs, whereas only trace amount of N can be identified for Co@CF/CNTs. By quantitative XPS analysis, the atomic percentages of N element in Co@N‐CF/CNTs and Co@CF/CNTs are 7.53% and 1.01% (Table S2, Supporting Information), respectively. The high‐resolution N 1s scan of Co@N‐CF/CNTs is displayed in Figure 3b and can be deconvoluted into five subpeaks. The three main peaks at 398.19, 399.02, and 400.74 eV are attributed to pyridinic N, pyrrolic N, graphitic N, respectively,[[qv: 5d,11b,20a,22]] whereas the small pair at the binding energy of 402.11 and 402.75 eV is assigned to oxidized N.[[qv: 5d,20a,23]] The schematic bonding structure of the four types of N dopants is shown in Figure 3g. The C 1s spectrum is shown in Figure 3c, and can be deconvoluted into three peaks at 284.6, 285.46, and 287.5 eV, which are attributed to graphitic sp^2^ C,[[qv: 11a,20a,24]] N—sp^2^ C, and N—sp^3^ C species,[[qv: 5d,11b,25]] respectively, further confirming the incorporation of N atoms into the carbon matrix. In contrast, no signal of N 1s (Figure 3b) was detected in Co@CF/CNTs, possibly due to the escape of nitrogen at higher annealing temperatures.[[qv: 11b]] The peak at 285.57 eV for Co@CF/CNTs (Figure S6a, Supporting Information) was ascribed to the formation of C—O bond during the annealing process.[[qv: 23a,24b,26]]

The full survey XPS of CoSe_2_@N‐CF/CNTs and CoSe_2_@CF/CNTs were shown in Figure 3d, where C, O, Co, and Se were clearly observed. As illustrated in Figure 3e, the binding energies of Co 2p_3/2_ and Co 2p_1/2_ for CoSe_2_@N‐CF/CNTs were identified at 778.5 and 793.3 eV, respectively, in accord with those of CoSe_2_.^27^ However, the binding energies of Co 2p_3/2_ and Co 2p_1/2_ for CoSe_2_@CF/CNT composite were observed at 780.9 and at 796.0 eV, implying the existence of oxidic Co,[[qv: 5d,28]] which might be ascribed to the partial surface oxidation of Co when exposed to air. On the other hand, the electron clouds have not been biased from CF to CoSe_2_ for CoSe_2_@CF/CNTs after Se incorporation owing to the absence of nitrogen dopant in carbon host.[[qv: 16a,29]] This perspective can be further proved by the much more intense peak of Se 3d electrons of CoSe_2_@CF/CNTs (Figure S6b, Supporting Information) at 59.2 eV than CoSe_2_@N‐CF/CNTs (Figure 3f) at 59.4 eV, which mainly originated from the intense interaction between Se and O atoms in the carbon matrix. In Figure 3f, the two peaks at 54.8 and 55.5 eV are assigned to Se 3d_5/2_ and 3d_3/2_ of CoSe_2_ of CoSe_2_@N‐CF/CNTs.[[qv: 11a,27a,b]]

### 2.2 Electrochemical Characterization

**Figure 4:**
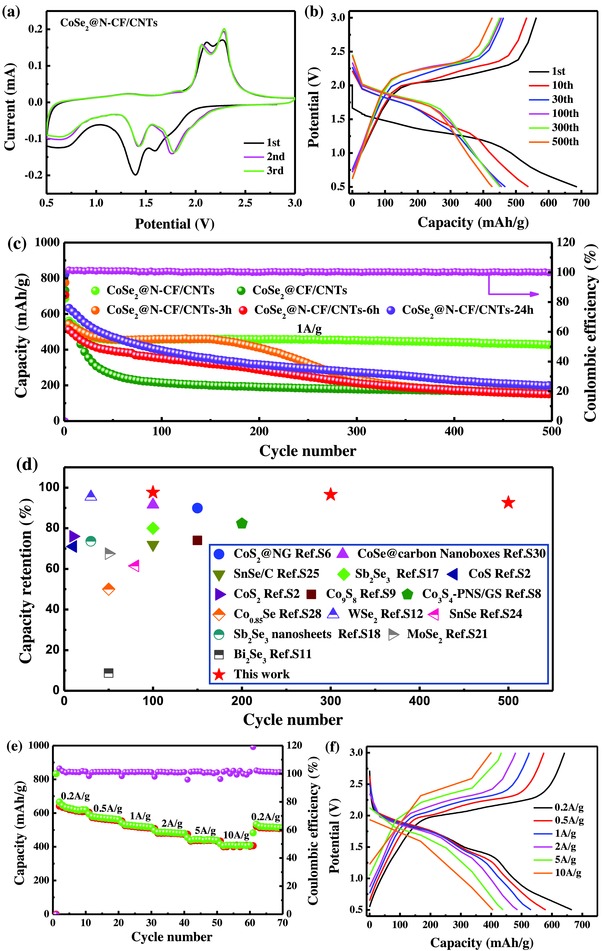
a) CV curves of CoSe_2_@N‐CF/CNTs at a scan rate of 0.2 mV s^−1^ in the voltage range of 3.0–0.5 V versus Li/Li^+^. b) Galvanostatic discharge and charge curves of CoSe_2_@N‐CF/CNTs at a current rate of 1 A g^−1^. c) Comparison of cycling performance of as‐prepared electrodes at 1 A g^−1^. d) Cycle life comparison of anode materials for LIBs in this work and previous literatures. e) Rate capability of CoSe_2_@N‐CF/CNTs at increasing current density from 0.2 to 10 A g^−1^. f) The corresponding charge/discharge profiles at various rates.

The electrochemical properties of the CoSe_2_@N‐CF/CNTs composite as an anode material for LIBs were then evaluated by using a 2032‐type coin cell assembled with a metallic lithium foil as the counter and reference electrode. The three initial cyclic voltammetry (CV) scans for CoSe_2_@N‐CF/CNTs were measured between 0.5 and 3.0 V at the scan rate of 0.2 mV s^−1^, as shown in **Figure** **4**a. The first CV curve displayed a much different shape from those of the following cycles. Specifically, the cathodic peaks at 1.59 and 1.39 V can be ascribed to the lithium insertion, corresponding to the sequential conversion of CoSe_2_ to Li*_x_*CoSe_2_,[[qv: 5a,30]] and then to metallic Co and Li_2_Se.[[qv: 18,30a–e]] Besides, the appearance of a weak and broad peak around 0.65 V is indicative of the inevitable formation of a solid electrolyte interface (SEI) film caused by the electrolyte decomposition and reaction on the anode surface.[[qv: 5a,18,30a–f,31]] In the anodic scan, two oxidation peaks at 2.11 and 2.27 V corresponded to the sequential phase transitions of the delithiation process.[[qv: 18,30a,b,32]] In the following CV scans, the cathodic peaks shifted toward the positive voltages, from 1.59 and 1.39 to 1.77 and 1.43 V, respectively, which probably originated from the electrochemical activation of the electrode materials in the 1st cycle.[[qv: 5a,18,30a,33]] Additionally, the second and third CV scans of CoSe_2_@N‐CF/CNTs are much better overlapped, as compared with CoSe_2_@CF/CNTs and other samples (Figure S7, Supporting Information), suggesting much more stable cycling performance of the former than the latter.

The galvanostatic discharge and charge profiles of the CoSe_2_@N‐CF/CNTs composite from 0.5 to 3.0 V at the current rate of 1 A g^−1^ are displayed in Figure 4b, and from the 1st cycle, the discharge and charge capacities were estimated to be 687 and 562 mAh g^−1^, with an initial Coulombic efficiency of 81.88%. Figure 4c shows the cycling performance of CoSe_2_@N‐CF/CNTs, CoSe_2_@CF/CNTs, and other samples at a current density of 1 A g^−1^. CoSe_2_@N‐CF/CNTs can be seen to deliver a reversible capacity of 428 mAh g^−1^ after 500 cycles with the Coulombic efficiency of almost 100%, corresponding to a high capacity retention of 92.58% and extremely slow capacity decay rate of 0.016% per cycle calculated from the 30th cycle. The enhanced cycling stability of CoSe_2_@N‐CF/CNTs, as compared to that of CoSe_2_@CF/CNTs, is probably due to the improved binding strength between CoSe_2_ and the N‐doped carbon matrix.[[qv: 11b,16a,34]] It is noted that the average crystal size of CoSe_2_@N‐CF/CNTs, with dwelling time of 12 h during selenization of Co@N‐CF/CNTs, is much bigger than those of other samples (Figure S8 and Table S3, Supporting Information), which might be equally responsible for the improved cycling performance of CoSe_2_@N‐CF/CNTs. The gravimetric capacity and cycling performance of CoSe_2_@N‐CF/CNTs and other anode materials for LIBs in recent literatures are summarized in Table S4 (Supporting Information) and compared in Figure 4d. It can be seen that the cycle stability of the CoSe_2_@N‐CF/CNTs electrodes is markedly better than that of other typical transition metal chalcogenide anodes, especially in the long charge/discharge process. The rate performance of CoSe_2_@N‐CF/CNTs is depicted in Figure 4e, with the specific capacity of 666, 580, 531, 487, 439, and 406 mAh g^−1^ at the current rate of 0.2, 0.5, 1, 2, 5, and 10 A g^−1^, respectively. Furthermore, when the current rate was reversed back to 0.2 A g^−1^, the composite recovered most of its specific capacity and reached a value of 534 mAh g^−1^. The capacity fading, compared to that of the initial few cycles, is probably due to the activation process similar to its cyclic behavior. To minimize the impact of the initial decay and evaluate the intrinsic rate performance of CoSe_2_@N‐CF/CNTs, the rate capability was further tested after first 30 cycles of activation at 1 A g^−1^ (Figure S9, Supporting Information). It can be seen that the capacity was almost completely recovered to the initial value when the current rate was reduced to 0.2 A g^−1^. Figure 4f depicts the corresponding charge/discharge voltage curves at the current density of 0.2, 0.5, 1, 2, 5, and 10 A g^−1^ of the CoSe_2_@N‐CF/CNTs electrode in the potential range of 0.5–3.0 V. Interestingly, even though the voltage gap was gradually enlarged with increasing current density, the voltage plateaus contributing to the charge and discharge capacities remain be obviously defined for all charge/discharge profiles, owing to the increasing polarization loss and mechanical energy dissipation, which indicates outstanding rate capability of CoSe_2_@N‐CF/CNTs.[[qv: 1c,5d,35]] As an optimizing process, the electrochemical performance of CoSe_2_@N‐CF/CNTs electrodes with ether‐based electrolyte and polyvinylidene fluoride (PVDF) binder were studied and shown in Figure S10 (Supporting Information). The inferior performance of CoSe_2_@N‐CF/CNTs electrodes here was attributed to the dissolution of the intermediate products into the ether‐based electrolyte^36^ and the lack of strong adhesion between the electrode layer and copper foil,^37^ respectively.

**Figure 5:**
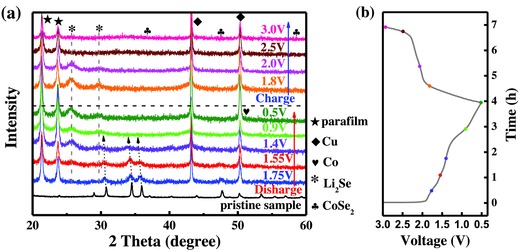
a) XRD patterns of the CoSe_2_@N‐CF/CNTs electrodes obtained at various charge–discharge states for the 1st cycle. b) The corresponding charge–discharge curve.

The mechanism for lithium‐ion storage was then investigated by means of ex situ XRD measurements. As shown in **Figure** **5**, the XRD patterns of CoSe_2_ continuously descended and shifted toward lower 2θ values when the cells was discharged to 1.75 and 1.55 V, which might be attributed to the intercalation of Li ions within the host CoSe_2_ materials, the formation of Li*_x_*CoSe_2_ intermediates, and the expansion of interlayer spacing, as observed previously for the intercalation of Li and Na ions into NiSe_2_, MoS_2_, and FeS_2_.[[qv: 30e,38]] When further discharged to 1.4, 0.9, and 0.5 V, the diffraction peaks of CoSe_2_ vanished and the diffraction peaks of Li_2_Se (111) and (200) lattice facets emerged at 2θ = 25.68° and 29.74° (PDF no. 23‐0072). The evolution of the XRD patterns during discharging process suggested the conversion of CoSe_2_ to Li*_x_*CoSe_2_ and further to Li_2_Se and Co. In the continuous charging process, the diffractions peaks of Li_2_Se can be clearly observed when charged back to 1.8 and 2.0 V, but completely disappeared when further charged to 2.5 and 3.0 V. This suggests that the conversion reactions happened between 2.0 and 2.5 V. Although CoSe_2_ failed to be detected in XRD measurements even if the electrode was fully charged to 3.0 V, ex situ Raman analysis clearly confirmed the A_g_ vibrational mode of CoSe_2_ at the fully charged state of 3.0 V in the 1st cycle (Figure S11, Supporting Information). The absence of CoSe_2_ in XRD measurements during the charging process might be attributed to the poor crystallinity of the electrochemically regenerated CoSe_2_,[[qv: 1c,3b,5d,38b,39]] as well as the interference of conductive additive, binder, and copper current collector.[[qv: 1c,40]] To recap, the structural evolution of the as‐prepared CoSe_2_@N‐CF/CNTs at different charge and discharge states is well consistent with the voltage curve and CV result. Based on the facts established above and from relevant literatures, the reaction mechanism of this composite can be described as follows:

In the discharge process

(1) $${\mathrm{CoSe}}_{2}+x{\mathrm{Li}}^{+}+xe^{-}\to {\mathrm{Li}}_{x}{\mathrm{CoSe}}_{2}$$

(2) $${\mathrm{Li}}_{x}{\mathrm{CoSe}}_{2}+{\mathrm{Li}}^{+}+e^{-}\to \mathrm{Co}+{\mathrm{Li}}_{2}\mathrm{Se}$$

In the charge process

(3) $$\mathrm{Co}+{\mathrm{Li}}_{2}\mathrm{Se}\to {\mathrm{Li}}_{x}{\mathrm{CoSe}}_{2}+{\mathrm{Li}}^{+}+e^{-}$$

(4) $${\mathrm{Li}}_{x}{\mathrm{CoSe}}_{2}\to {\mathrm{CoSe}}_{2}+x{\mathrm{Li}}^{+}+xe^{-}$$

Total reaction equation

(5) $${\mathrm{CoSe}}_{2}+4{\mathrm{Li}}^{+}+4e^{-}↔\mathrm{Co}+2{\mathrm{Li}}_{2}\mathrm{Se}$$

**Figure 6:**
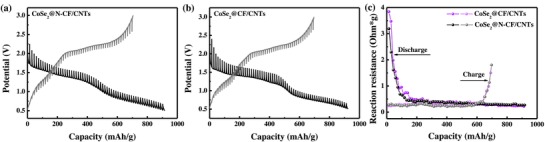
a,b) GITT voltage profiles and c) reaction resistances of CoSe_2_@N‐CF/CNTs and CoSe_2_@N‐CF/CNTs electrodes during the first lithiation and delithiation processes.

Galvanostatic intermittent titration technique (GITT) was employed to evaluate ion insertion and extraction levels and the electrochemical reaction kinetics of the as‐prepared electrodes. **Figure** **6**a,b plotted the potential response curves of CoSe_2_@N‐CF/CNTs and CoSe_2_@CF/CNTs for both lithiation and delithiation processes during the 1st cycle, in which the overpotential looks stable and remains at a very low level. Furthermore, the CoSe_2_@N‐CF/CNTs electrode exhibited slightly lower reaction resistances for both Li^+^ insertion and extraction processes than CoSe_2_@CF/CNTs, indicating the improved lithium‐ion diffusion kinetics and explaining well the superior electrochemical performance of the CoSe_2_@N‐CF/CNTs composite.[[qv: 23a,41]]

**Figure 7:**
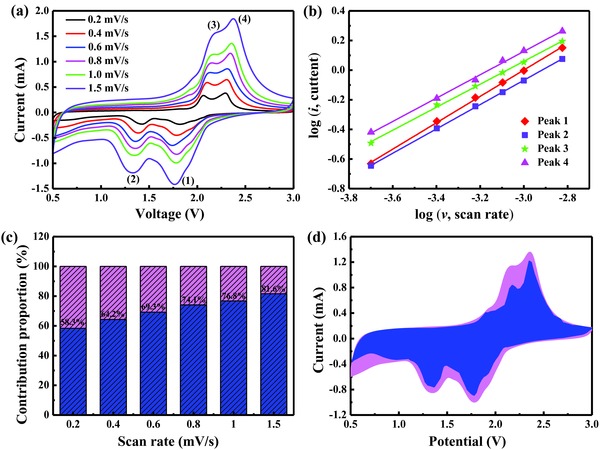
a) CV curves of CoSe_2_@N‐CF/CNTs for Li‐ion storage with scan rates from 0.2 to 1.5 mV s^−1^. b) The corresponding plots of log(*i*) versus log(*v*) at each redox peak (peak current: *i*, scan rate: *v*). c) Normalized percentage of pseudocapacitance (blue) at different scan rates. d) The pseudocapacitive contribution (blue region) to the total current at a scan rate of 1.0 mV s^−1^.

To further explain the excellent rate performance of the CoSe_2_@N‐CF/CNTs electrode, the redox kinetics was investigated by CV measurements to separate the pseudocapacitance‐like capacity and diffusion‐controlled contribution. **Figure** **7**a exhibits similar CV profiles at sweep rates from 0.2 to 1.5 mV s^−1^, where two pairs of cathodic and anodic peaks distinctly exist. As shown in Figure S12 (Supporting Information), the nonlinear correlation between the peak current (*i*) and the square root of the scan rate (*v*) suggested that Li storage includes both Faradaic and non‐Faradaic contributions.[[qv: 1c,42]] According to previous researches,[[qv: 1c,6c,42,43]] the current response and the scan rate obey the described relationship

(6) $$i=av^{b}$$

(7) $$\log\left(i\right)=b\times \log\left(v\right)+\log\left(a\right)$$

where *a* and *b* express adjustable parameters. At *b* = 0.5, the diffusion‐controlled behavior is prevailing during the charge/discharge process, whereas at *b* = 1, pseudocapacitive effect dominated.[[qv: 1c,6c]] In the current study, the *b*‐values of the two reduction peaks (peaks 1 and 2) and the corresponding oxidation peaks (peak 3 and 4) were determined to be 0.89, 0.82, 0.77, and 0.79 by log(*i*) versus log(*v*) plots (Figure 7b), respectively, indicating that the redox kinetic process of CoSe_2_@N‐CF/CNTs composite consisted of both diffusion‐controlled and pseudocapacitance behaviors. Furthermore, the pseudocapacitance‐like contribution at a specific scan rate can be quantified by the following equation

(8) $$i=k_{1}v+k_{2}v^{1/2}$$

in which *k*_1_ *v* and *k*_2_ *v* ^1/2^ are the pseudocapacitive capacity and diffusion‐controlled capacity, respectively. As summarized in Figure 7c, the pseudocapacitive contribution was estimated to be 58.3%, 64.2%, 69.3%, 74.1%, 76.8%, and 81.6% at the scan rates of 0.2, 0.4, 0.6, 0.8, 1.0, and 1.5 mV s^−1^, respectively, showing a continuous increase with increasing scan rate. As expected, the pseudocapacitive contribution dominates the charge‐storage capacity at high scan rates, which is beneficial for fast Li^+^ transfer kinetics during the intercalation/extraction process.[[qv: 1c,42]] In addition, Figure 7d further reveals the detailed pseudocapacitive portion (blue region) in comparison with the total current measured at a scan rate of 1.0 mV s^−1^.

Moreover, the CoSe_2_@N‐CF/CNTs electrode investigated under the cut‐off voltage range of 0.01–3.0 V (Figure S13, Supporting Information) also exhibits high reversible capacity and robust rate capability. As seen in Figure S13c (Supporting Information), the CoSe_2_@N‐CF/CNTs electrode delivered a reversible capacity of 1629, 1508, and 1006 mAh g^−1^ in the 100th cycle at current rates of 0.2, 0.5, and 1 A g^−1^, respectively. In particular, the specific capacity decreased first and then increased gradually from the 8th cycle, which was normally related to the accelerated Li^+^ diffusion kinetics by a progressive activation behavior, the gradual formation of electroactive polymeric gel‐like layers, and the accumulation of the interfacial substances, such as Li, LiOH, Li_2_O, and LiH.[[qv: 3a,5a,b,44]] As shown in Figure S13d,e (Supporting Information), the discharge capacity slightly reduced from 1064 to 1039, 1031, 1006, 976, and 864 mAh g^−1^ with increasing current density from 0.1 to 0.2, 0.5, 1, 2, and 5 A g^−1^, suggesting its very small polarization and excellent rate performance.

**Figure 8:**
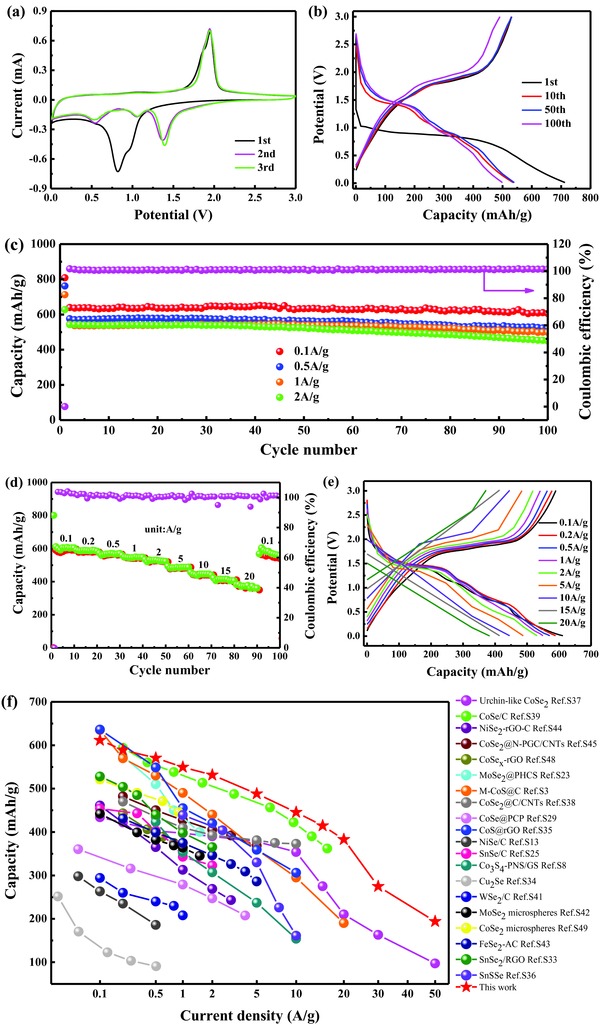
Electrochemical performances of CoSe_2_@N‐CF/CNTs as an anode for SIBs. a) CV curves in the voltage range of 3.0–0.01 V versus Na/Na^+^ at a scan rate of 0.2 mV s^−1^. b) Galvanostatic discharge and charge profiles at a current density of 1 A g^−1^. c) Comparison of cycling performance at the current rates of 0.1, 0.5, 1, and 2 A g^−1^. d) Rate capability at increasing current density from 0.1 to 20 A g^−1^. e) The corresponding galvanostatic charge/discharge curves at various rates. f) Comparison of rate capability of the produced CoSe_2_@N‐CF/CNTs with other typical anode materials for SIBs.

Since the size of Na and Li ions are different, very limited materials are available as high‐performance electrodes for both LIBs and SIBs. To corroborate the promising practicability of the CoSe_2_@N‐CF/CNTs composites for energy storage, SIBs were also fabricated to evaluate the electrochemical properties for Na‐storage. **Figure** **8**a shows the CV profiles of the SIBs with CoSe_2_@N‐CF/CNTs as anode materials at a scan rate of 0.2 mV s^−1^, where a shoulder and a main peak at 0.95 and 0.82 V can be observed in the first cathodic scan, due to the reaction of metalloid Se to Na_2_Se[[qv: 36b,45]] and the conversion reaction of CoSe_2_ to Co and Na_2_Se, respectively.[[qv: 2b,45]] Correspondingly, the weak shoulder and peak at 1.88 and 1.95 V in the first anodic scan might be assigned to the recuperation of CoSe_2_ from metallic Co and Na_2_Se.[[qv: 1c,2b,8,45]] In the subsequent CV sweeps, three major reduction peaks emerged at 1.36, 1.06, and 0.55 V, respectively, corresponding to sodiation reactions as described by Equations (9)–(11), respectively,[[qv: 1c,2b,8,45]] while the anodic oxidation peak overlapped very well with that of the first scan, which could be described by Equation (12). The electrochemical reaction of CoSe_2_@N‐CF/CNTs for Na‐storage can be summarized as follows:

In the discharge process

(9) $${\mathrm{CoSe}}_{2}+x{\mathrm{Na}}^{+}+xe^{-}\to {\mathrm{Na}}_{x}{\mathrm{CoSe}}_{2}$$

(10) $${\mathrm{Na}}_{x}{\mathrm{CoSe}}_{2}+\left(2-x\right){\mathrm{Na}}^{+}+\left(2-x\right)e^{-}\to \mathrm{CoSe}+{\mathrm{Na}}_{2}\mathrm{Se}$$

(11) $$\mathrm{CoSe}+2{\mathrm{Na}}^{+}+2e^{-}\to \mathrm{Co}+{\mathrm{Na}}_{2}\mathrm{Se}$$

In the charge process

(12) $$\mathrm{Co}+2{\mathrm{Na}}_{2}\mathrm{Se}\to {\mathrm{CoSe}}_{2}+4{\mathrm{Na}}^{+}+4e^{-}$$

CoSe_2_@N‐CF/CNTs displayed the discharge and charge capacities of 713 and 531 mAh g^−1^ in the 1st cycle at a current density of 1 A g^−1^ with Coulombic efficiency of 74.53%, as shown in Figure 8b. The cycling performance of CoSe_2_@N‐CF/CNTs at current densities of 0.1, 0.5, 1, and 2 A g^−1^ was exhibited in Figure 8c. It can be seen that the discharge capacity at a current density of 0.1 A g^−1^ decreases slightly from 639 mAh g^−1^ in the 2nd cycle to 606 mAh g^−1^ in the 100th cycle with a remarkable capacity retention of 94.74%. When cycled at current rates of 0.5, 1, and 2 A g^−1^, CoSe_2_@N‐CF/CNTs delivered discharge capacities of 523, 499, and 450 mAh g^−1^ after 100 cycles, with capacity retentions of 90.69%, 91.78%, and 83.03% with the respect to that of the 2nd cycle. Such excellent cycling stability of CoSe_2_@N‐CF/CNTs can be ascribed to its robust structure. As exhibited in Figure S14 (Supporting Information), the overall structural morphology of the CoSe_2_@N‐CF/CNTs composite after 100 cycles was well maintained, indicating its good structural integrity. Additionally, the CoSe_2_@N‐CF/CNTs electrode with a CoSe_2_ loading of 2.0 mg cm^−2^ yields a high specific capacity of 449 mAh g^−1^ at the current density of 1 A g^−1^ (Figure S15, Supporting Information), further demonstrating the superior structural advantages. By contrast, pure N‐CF/CNTs obtained by etching the inner Co nanoparticles delivered very low capacity after 100 cycles (Figure S16, Supporting Information), suggesting the dominant contribution of CoSe_2_ in the composite electrode.

**Figure 9:**
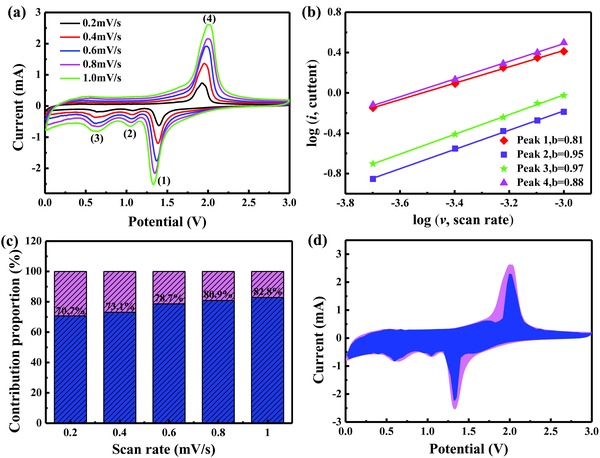
Electrochemical kinetics analysis of CoSe_2_@N‐CF/CNTs for Na‐ion storage. a) CV curves at different scan rates. b) The corresponding log(*i*) versus log(*v*) plots at each redox peak (peak current: *i*, scan rate: *v*). c) Normalized contribution percent of pseudocapacitive‐controlled capacity (blue) at different scan rates. d) The pseudocapacitive contribution (blue region) to the total current at a scan rate of 1 mV s^−1^.

As depicted in Figure 8d,e, a high and stable discharge capability can be preserved at different current densities for Na‐ion storage, specifically, 612, 589, 571, 550, 531, 488, 446, 415, and 383 mAh g^−1^ at 0.1, 0.2, 0.5, 1, 2, 5, 10, 15, and 20 A g^−1^. More strikingly, except for enlarged polarization, reversible capacities of 275 and 194 mAh g^−1^ were still obtained even at ultrahigh current rates of 30 and 50 A g^−1^ (Figure S17, Supporting Information), demonstrating its excellent rate stability and bright application prospect. Similarly, the electrochemical properties of CoSe_2_@N‐CF/CNTs were equally compared with other anode materials for SIBs reported in recent literatures and summarized in Table S5 (Supporting Information). The gravimetric capacity and cycle stability of CoSe_2_@N‐CF/CNTs in the current work excelled many anode materials reported in the literature. Importantly, compared to other metal sulfides/selenides, the CoSe_2_@N‐CF/CNTs showed prominent advantages in rate capability especially at high current density (Figure 8f). Similar to LIBs, the pseudocapacitive contributions to the capacity of CoSe_2_@N‐CF/CNTs as anode materials for SIBs were analyzed, which were calculated to be 70.7%, 73.1%, 78.7%, 80.9%, and 82.8% at the scan rates of 0.2, 0.4, 0.6, 0.8, and 1 mV s^−1^, respectively, as shown **Figure** **9**. The pseudocapacitive contribution to the reversible capacity was higher for SIBs than for LIBs, which might account for the higher rate capability of the former than the latter.

The remarkable electrochemical performance of CoSe_2_@N‐CF/CNTs composite was also confirmed by electrochemical impedance spectroscopy (EIS) measurements, which were conducted on the electrodes before and after cycling in the frequency range of 100 kHz–100 mHz at the fully charged state. As shown in Figure S18 (Supporting Information), the interfacial charge transfer resistance (*R*_ct_) retained a very small value after initial activation and even long cycling. In fact, *R*_ct_ for LIBs was only 35 and 33 Ω after 100 cycles in the cut‐off voltage range of 0.5–3.0 and 0.01–3.0 V, respectively; and *R*_ct_ remained at 30 Ω after 100 cycles for SIBs, indicating structural stability of the CoSe_2_@N‐CF/CNTs during the repeated charge and discharge processes.[[qv: 2b,45]] Such low and stable *R*_ct_ not only guarantees fast charge transfer but also favors efficient Li^+^/Na^+^ insertion/desertion, which is beneficial for long cycle stability and high rate capability.[[qv: 1c,3b,8]] Furthermore, we conducted self‐discharge test to elaborate the advantageous electrochemical behavior of the CoSe_2_@N‐CF/CNTs electrode. The changes in the open circuit voltage (OCV) with measured time were depicted in Figure S19 (Supporting Information). The OCV fell slightly from 3.203 to 3.163 V for LIBs after 139 h, and from 2.767 to 2.758 V after 69 h for SIBs, indicating the steady interfacial characteristics and the teeny cell polarization.^46^

The carbon framework of CoSe_2_@N‐CF/CNTs offers sufficient space to accommodate the redox reaction products and thus effectively buffers the volume changes during cycling.[[qv: 2b,3a,6c]] Besides, doping nitrogen heteroatoms into the carbon architecture can introduce more active sites and enhance the electronic conductivity, thus improving the electrochemical performance.[[qv: 6b,47]] Furthermore, a large number of external‐growth CNTs are of great importance to prevent the agglomeration of electrochemistry active component and the electrode pulverization, thus ensuring the structural integrity.[[qv: 1d,5a]] The omnibearing conductive network, generating from the N‐CF skeleton and the surficial interconnected CNTs, can not only increase electrolyte/electrode contact and facilitate electrolyte infiltration, but also provide rapid diffusion paths for electrons/ions transfer, resulting in enhanced reaction kinetics.[[qv: 1d,3a,8,9]] As a consequence, these superior structure properties confer CoSe_2_@N‐CF/CNTs with high reversible capacity, exceptional rate capability, and long cycling life when applied as dual‐role anode materials for both LIBs and SIBs.

## 3 Conclusion

In summary, hybrid CoSe_2_@N‐CF/CNTs superstructures, comprised of inner CoSe_2_ nanoparticles and outer CNT‐intertwined N‐doped carbon framework, have been prepared from ZIF‐67 by a facile two‐step heat treatment procedure. Benefiting from the unique structure, the CoSe_2_@N‐CF/CNTs composite exhibited superior electrochemical performance as dual anode materials for both LIBs and SIBs. Specifically, it delivered a high reversible capacity of 428 mAh g^−1^ at 1 A g^−1^ even after 500 cycles for LIBs and 606 mAh g^−1^ at 0.1 A g^−1^ after 100 cycles for SIBs. Besides, electrochemical kinetics analysis indicated that the pseudocapacitive contribution dominates the charge‐storage capacity at high scan rates, accounting for high rate performance for both LIBs and SIBs. The effective synthesis strategy described in this current work provided new insights to the construction of high‐performance dual anode materials for both LIBs and SIBs.

## 4 Experimental Section

*Synthesis of ZIF‐67 Crystal*: All chemical reagents were of analytical grade and directly used without further purification. In a typical procedure, 16 mmol (1.312 g) of 2‐methylimidazole (Sigma‐Aldrich) was dissolved in 50 mL of methanol to form a clear solution, which was subsequently injected into 50 mL of methanol containing 4 mmol (1.164 g) of Co(NO_3_)_2_ · 6H_2_O (Damao Chemical Reagents Factory). The solution was thoroughly mixed after vigorous stirring for 1 h and then incubated at room temperature for 24 h. The as‐obtained precipitate was collected by centrifugation, repeatedly washed with methanol for at least 3 times, and then dried in a vacuum oven at 70 °C overnight to obtain purple ZIF‐67 crystals.

*Synthesis of Co@N‐CF/CNTs and Co@CF/CNTs*: The ZIF‐67 crystal prepared above was put into a ceramic boat and then directly annealed for 3 h in a horizontal tube furnace under an Ar/H_2_ gas flow (10% H_2_), at a heating rate of 2 °C min^−1^. Annealing at 700 °C of the ZIF‐67 precursor resulted in the formation of a carbon framework heavily doped with nitrogen, which was denoted as Co@N‐CF/CNTs. In contrast, the carbon framework obtained by annealing the ZIF‐67 precursor at 900 °C for 3 h contained no nitrogen doping and was denoted as Co@CF/CNTs.

*Synthesis of CoSe_2_@N‐CF/CNTs and CoSe_2_@CF/CNTs*: Commercial Se powders and the Co@N‐CF/CNTs composites prepared above were thoroughly ground at a mass ratio of 1:1 in a quartz mortar for 1 h and then annealed under an argon atmosphere at 300 °C for 3, 6, 12, and 24 h, at a heating rate of 2 °C min^−1^. The obtained products were designated as CoSe_2_@N‐CF/CNTs‐3h, CoSe_2_@N‐CF/CNTs‐6h, CoSe_2_@N‐CF/CNTs, and CoSe_2_@N‐CF/CNTs‐24h, respectively. As a control, CoSe_2_@CF/CNTs was also synthesized in the same manner except that Co@CF/CNTs were used instead.

*Material Characterization*: XRD measurements were performed on the Bruker D8 Advance (Germany) using Cu‐Kα radiation (λ = 1.5406 Å). TGA was conducted on Mettler Toledo TGA/SDTA851 in an O_2_ atmosphere at the heating rate of 10 °C min^−1^. Nitrogen adsorption–desorption isotherms were acquired at 77 K with an Autosorb‐iQ automatic volumetric instrument. Raman spectra were obtained at a wavelength of 514 nm using a high‐resolution dispersive Raman spectroscopy (Horiba JobinYvon, ARAMIS). XPS were acquired with a Phi X‐tool instrument. The microstructure and morphology of the samples were examined by field‐emission scanning electron microscopy (FESEM, Hitachi S‐4800) and HRTEM (JEOL, JEM‐2010).

*Electrochemical Measurements*: The working electrode was prepared by a slurry coating procedure. First, the active material, carbon black (Super P, Timcal), and water‐soluble binder sodium alginate ((C_6_H_7_O_6_Na)*_x_*, SA) were mixed at a mass ratio of 8:1:1 to obtain a homogeneous slurry. Then, the slurry was evenly pasted onto a copper foil using a film applicator, and dried at 70 °C in an electric oven overnight to remove the solvent. The mass loading of active materials was estimated to be 1.5–1.8 mg cm^−2^ for each electrode.

CR2032‐type coin cells were assembled in an argon‐filled glove box (Vigor‐LG2400/750TS, LTD, Suzhou), in which the oxygen and water contents were <1 ppm. During the assembly process, the Li metal of 15.6 × 0.45 mm in size was used as both the counter and reference electrodes, with a Celgard‐2400 film as the separator and 1.0 m LiPF_6_ in a mixed solvent of ethylene carbonate (EC), ethyl methyl carbonate (EMC), and dimethyl carbonate (DMC) at a volume ratio of 1:1:1 as the electrolyte. As for sodium battery, a Na foil was used as the counter and reference electrode, and Whatman GF/D was used as the separator. The electrolyte was 1.0 m NaClO_4_ in a mixed solvent of EC and DMC (1:1, vol%) with 5.0 wt% of fluoroethylene carbonate (FEC). CV and EIS measurements were carried out with a CHI 660 electrochemical workstation (Shanghai CH Instrument Co., Ltd.) at room temperature. The galvanostatic charge/discharge measurements were conducted with a multichannel battery testing system (CT2001A, LAND). For GITT measurement, the coin cells were first charged or discharged at a constant current pulse of 100 mA g^−1^ for 10 min, followed by an equal duration relaxation of 1 h, allowing the equilibrium potential of lithium storage at different points to be probed in the whole voltage window of 0.5–3.0 V.

## Conflict of Interest

The authors declare no conflict of interest.

## Supporting information

Supplementary
