# Supplementary material for: CoSe2 Nanoparticles Encapsulated by N‐Doped Carbon Framework Intertwined with Carbon Nanotubes: High‐Performance Dual‐Role Anode Materials for Both Li‐ and Na‐Ion Batteries
Source: Adv Sci (Weinh). 2018 Oct 17;5(12):1800763. doi: 10.1002/advs.201800763 (PMC6299709; doi:10.1002/advs.201800763)
Supplement: Supplementary file 1 — Supplementary [file ADVS-5-1800763-s001.pdf]

Copyright WILEY-VCH Verlag GmbH & Co. KGaA, 69469 Weinheim, Germany, 2018.

ADVANCED SCIENCE
Open Access

Supporting Information

for *Adv. Sci.,* DOI: 10.1002/advs.201800763

$CoSe_2$ Nanoparticles Encapsulated by N-Doped Carbon Framework Intertwined with Carbon Nanotubes: High-Performance Dual-Role Anode Materials for Both Li- and Na-Ion Batteries

Jun Yang, Hongcheng Gao, Shuang Men, Zhenqing Shi, Zhang Lin, Xiongwu Kang, and Shaowei Chen**

Supporting Information

$CoSe_2$ Nanoparticles Encapsulated by N-doped Carbon Framework Intertwined with Carbon Nanotubes: High-Performance Dual-Role Anode Materials for both Li and Na Ion Batteries

Jun Yang[a], Hongcheng Gao[a], Shuang Men[a], Zhenqing Shi[b], Zhang Lin[b], Xiongwu Kang*[a] and Shaowei Chen*[a,c]

[a] Guangzhou Key Laboratory for Surface Chemistry of Energy Materials, New Energy Research Institute, School of Environment and Energy, South China University of Technology, Guangzhou 510006, China
[b] Guangdong Engineering and Technology Research Center for Environmental Nanomaterials, School of Environment and Energy, South China University of Technology, Guangzhou, Guangdong 510006, China
[c] Department of Chemistry and Biochemistry, University of California, 1156 High Street, Santa Cruz, California 95064, United States
E-mails: esxkang@scut.edu.cn; shaowei@ucsc.edu

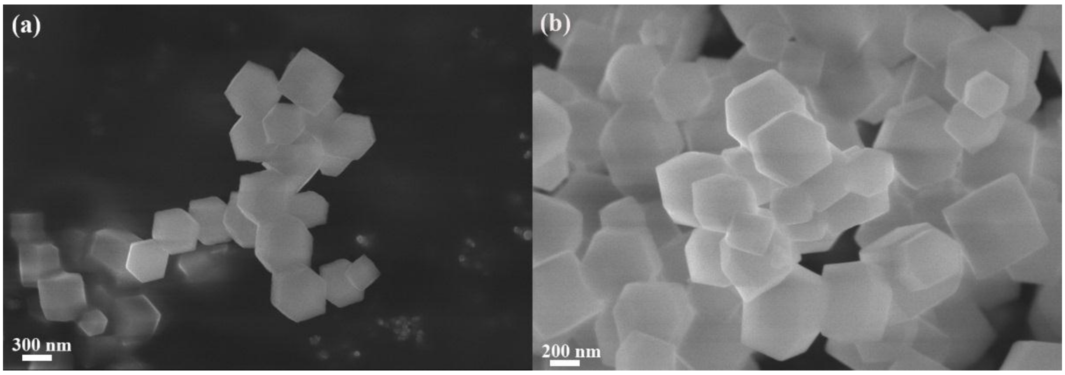

Fig. S1 SEM images of as-prepared ZIF-67.

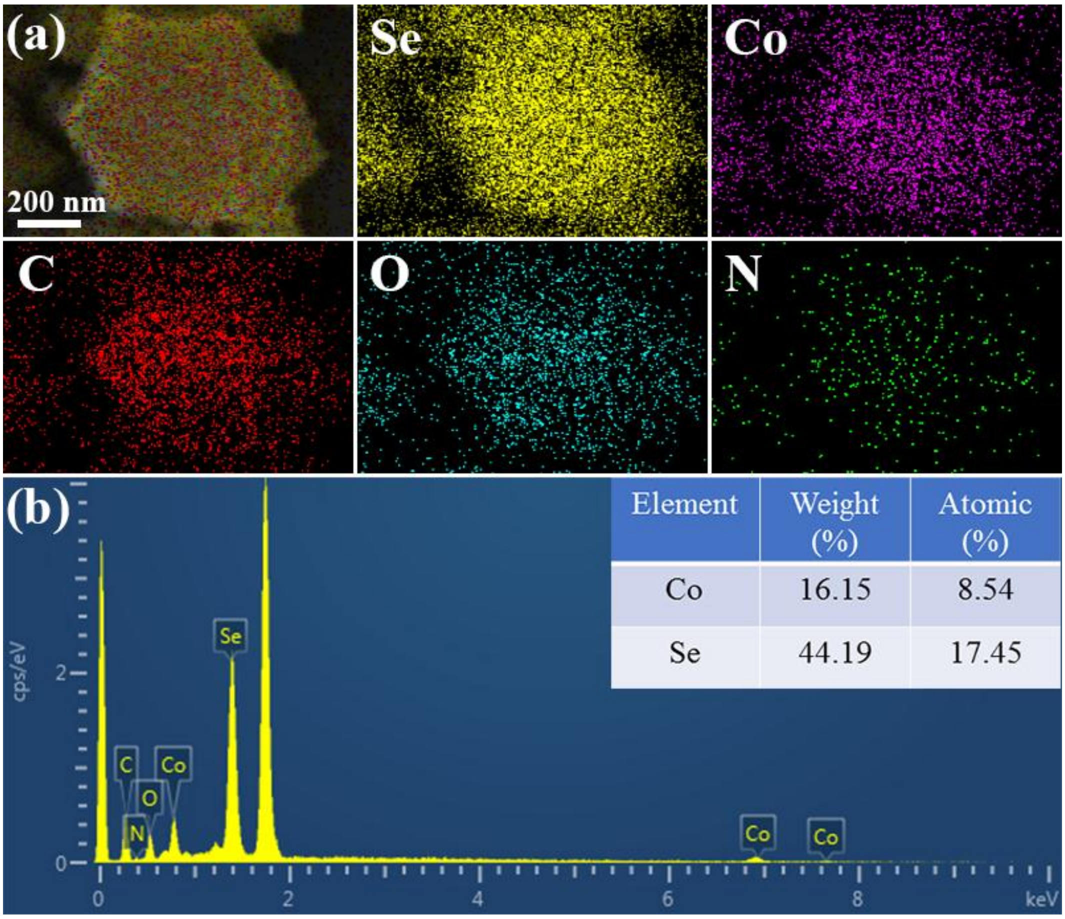

Fig. S2 (a) EDX elemental mapping of Se, Co, C, O and N and (b) EDX spectrum of CoSe$_2$@N-CF/CNTs composite. Inset to (b) is the summary of the Co and Se atomic fractions.

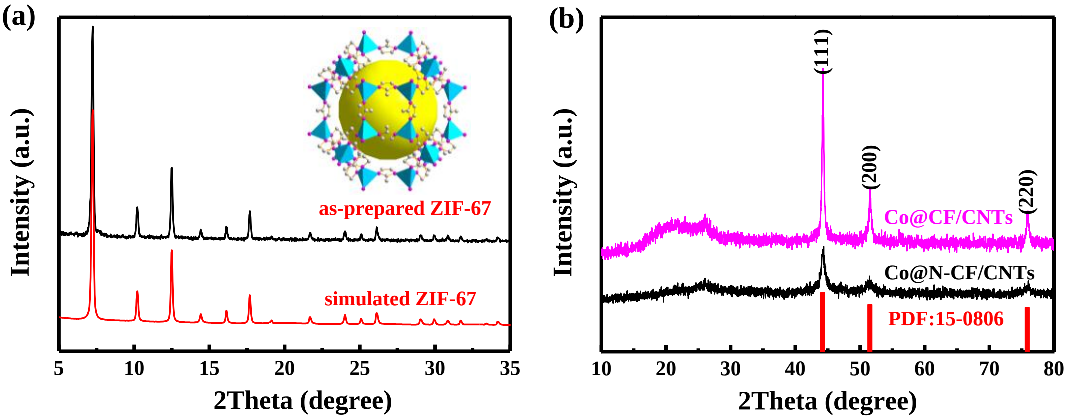

Fig. S3 XRD patterns of (a) as-prepared ZIF-67 and simulated ZIF-67, and (b) Co@N-CF/CNTs and Co@CF/CNTs. The inset to panel (a) reveals the sodalite (SOD) topology.

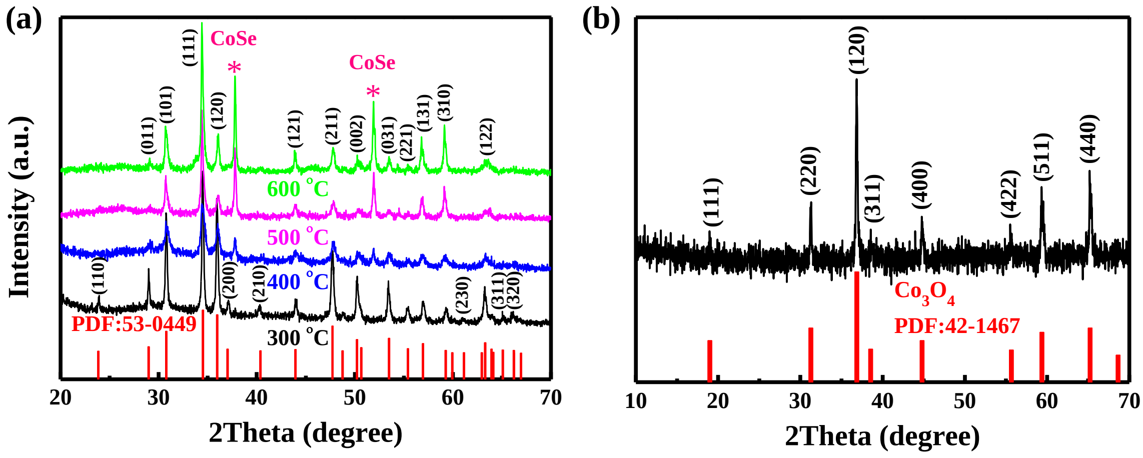

Fig. S4 (a) XRD patterns of the Co@N-CF/CNTs composites annealed with Se powder at different 300, 400, 500 and 600 °C for 12 hours. The diffraction peaks at 2θ = 37.76° and 51.91° were indexed to cubic CoSe (PDF No. 09-0234; Pa3(205), a = 5.843 Å, b = 5.843 Å, c =5.843 Å), while the temperature drops to 300 °C, a pure phase of orthorhombic $CoSe_2$ (PDF No. 53-0449; Pnnm(58), a = 3.643 Å, b = 4.896 Å, c =5.821 Å) can be obtained. (b) XRD pattern of the product Co_3O_4 after TGA measurement in air flow. During the TGA test process in air flow, the carbon is burned out, while the $CoSe_2$ was oxidized to Co_3O_4. According to chemical reaction $3CoSe_2$ (s) + $2O_2$ (g) = Co_3O_4 (s) + 6Se (g), the contents of $CoSe_2$ in $CoSe_2$@N-CF/CNTs and $CoSe_2$@CF/CNTs were calculated to be about 65.4 wt% and 68.6 wt%, respectively.

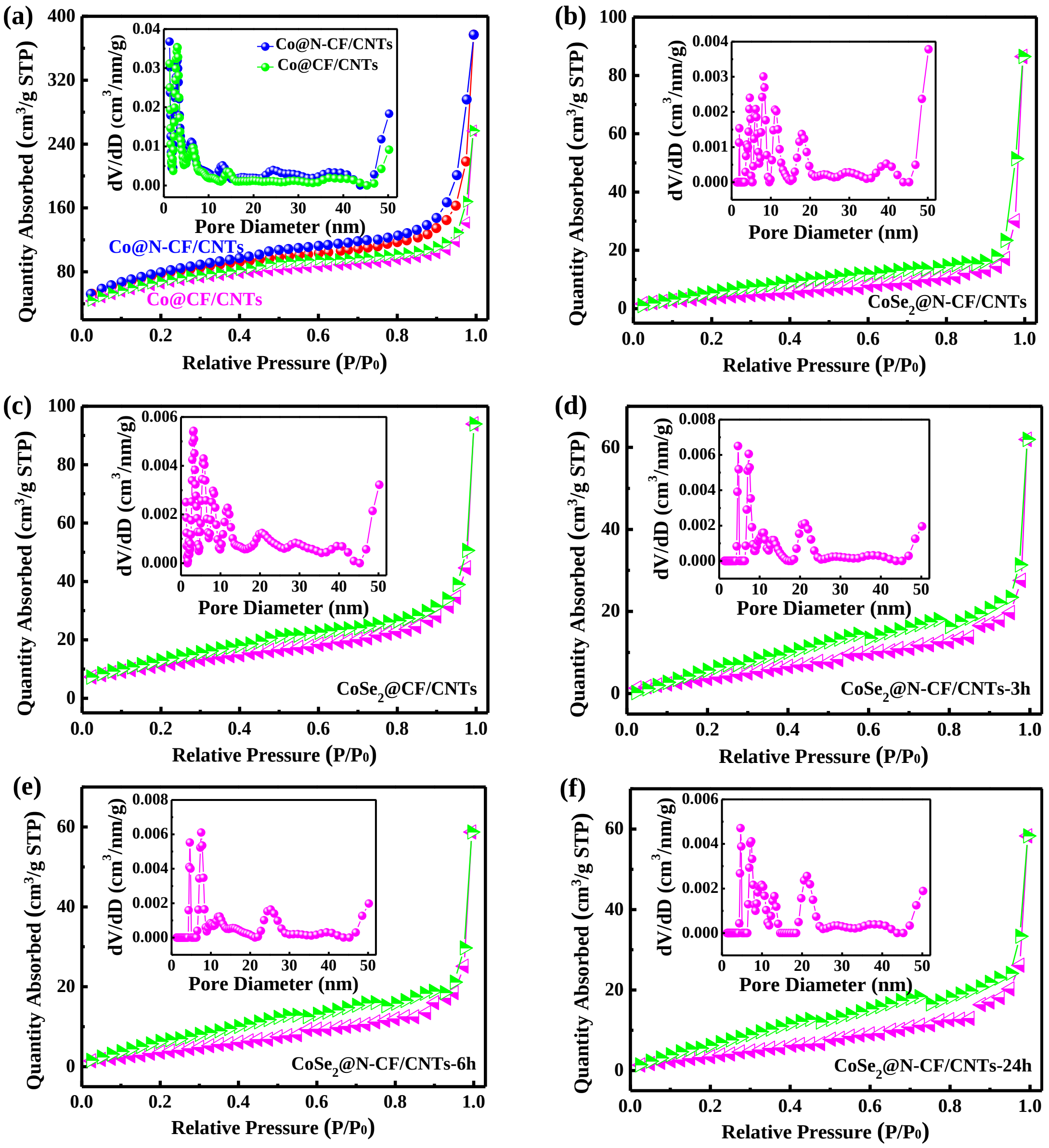

Fig. S5 Nitrogen adsorption-desorption isotherms of (a) Co@N-CF/CNTs and Co@CF/CNTs, (b) $CoSe_2$@N-CF/CNTs, (c) $CoSe_2$@CF/CNTs, (d) $CoSe_2$@N-CF/CNTs-3h, (e) $CoSe_2$@N-CF/CNTs-6h, and (f) $CoSe_2$@N-CF/CNTs-24h. Insets are the corresponding pore size distribution plots calculated by using the DFT method.

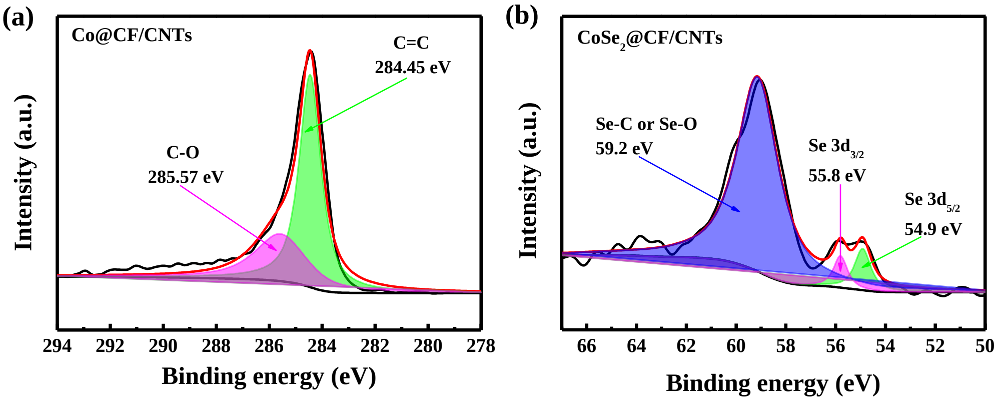

Fig. S6 High-resolution scans of (a) C 1s and (b) Se 3d electrons of Co@CF/CNTs.

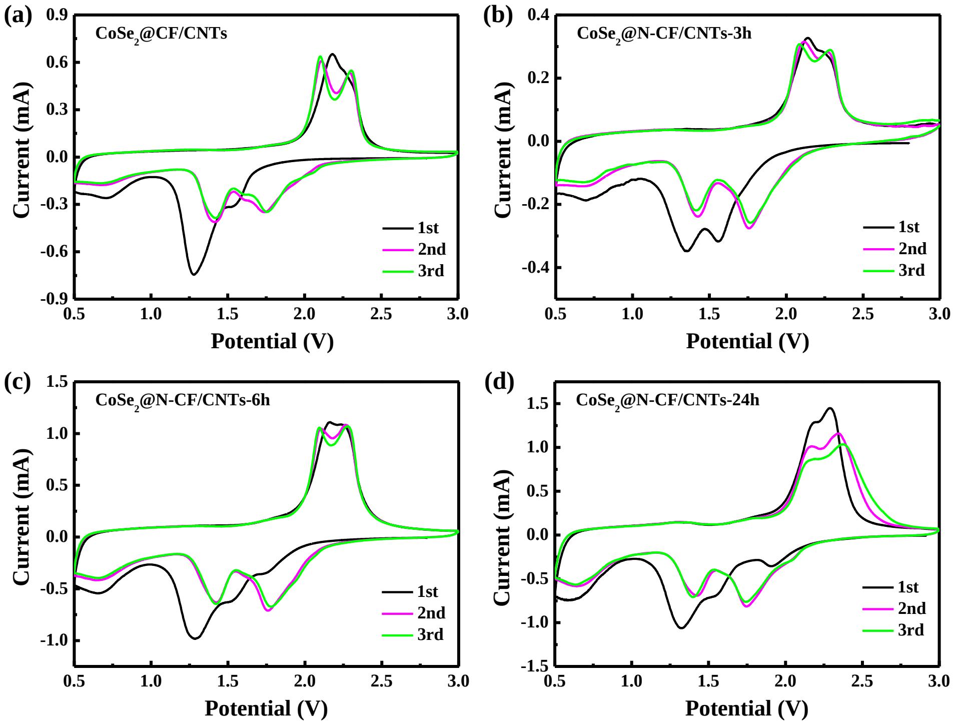

Fig. S7. CV curves of (a) $CoSe_2$@CF/CNTs, (b) $CoSe_2$@N-CF/CNTs-3h, (c) $CoSe_2$@N-CF/CNTs-6h, and (d) $CoSe_2$@N-CF/CNTs-24h at a scan rate of 0.2 $mV \cdot s^{-1}$ in the voltage range of 3.0-0.5 V vs Li/Li^+.

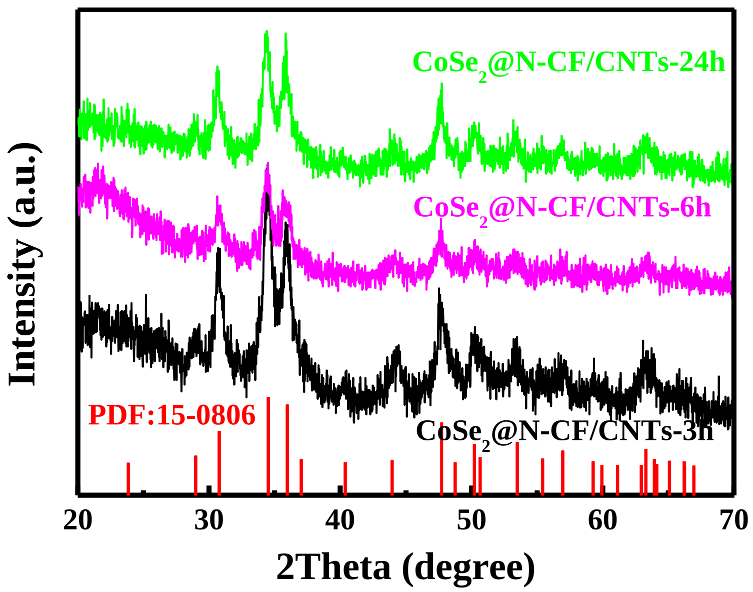

Fig. S8 XRD patterns of $CoSe_2$@N-CF/CNTs composites synthesized at different dwelling time at 300 °C.

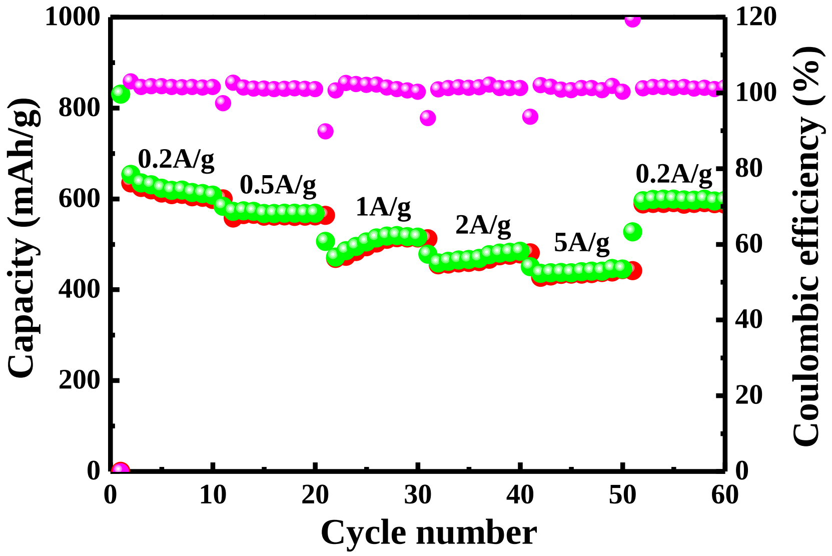

Fig. S9 Rate performance of the $CoSe_2$@N-CF/CNTs tested after first 30 cycles of activation at 1 A/g.

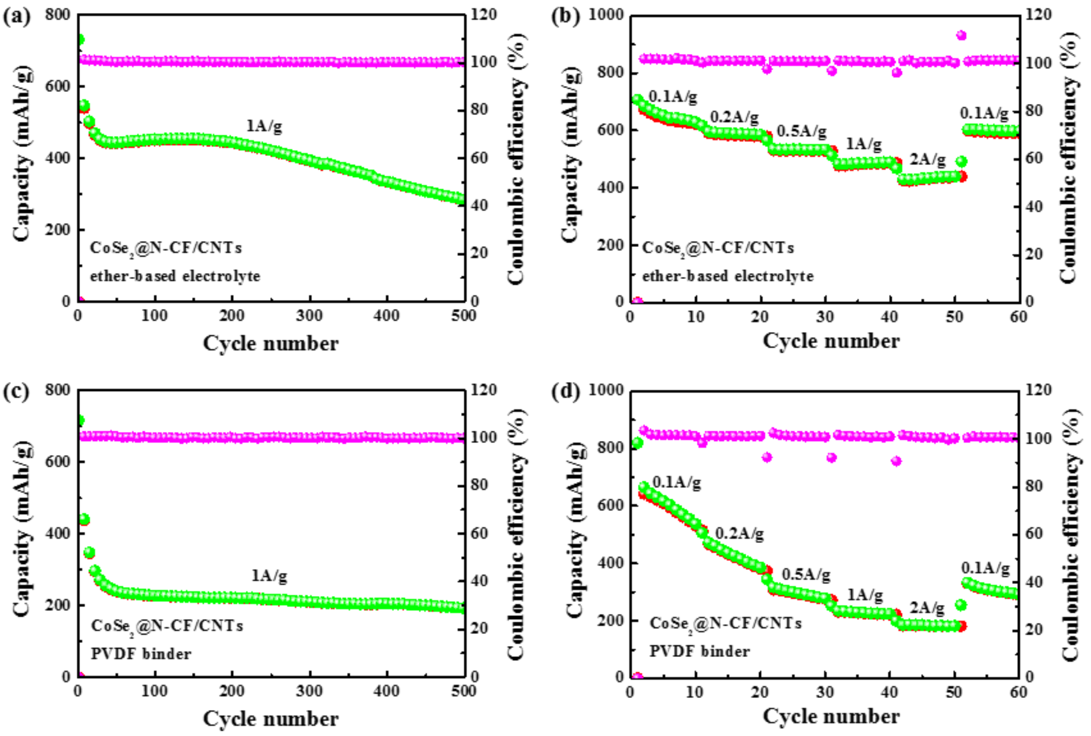

Fig. S10 Electrochemical performance of the as-prepared $CoSe_2$@N-CF/CNTs (a, b) with ether-based electrolyte (1M LiTFSI in DME/DOL (v/v = 1:1) with 1wt% of $LiNO_3$), and (c, d) with the PVDF binder.

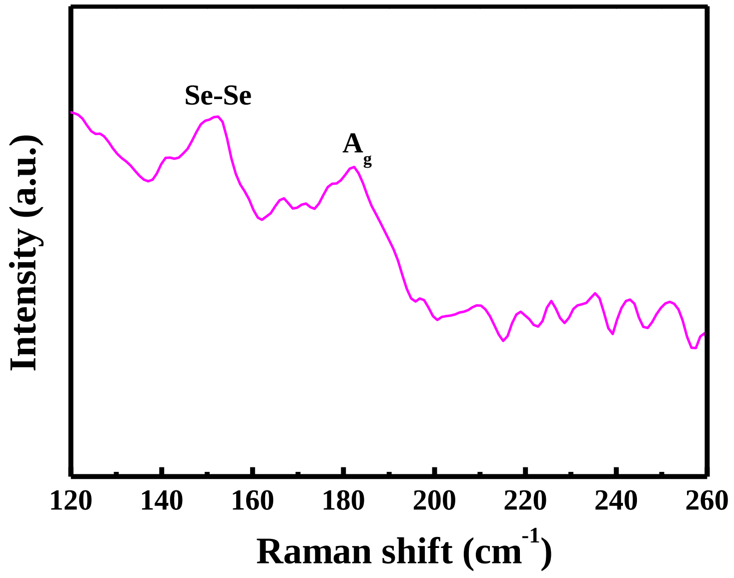

Fig. S11 Raman spectrum of the $CoSe_2$@N-CF/CNTs electrode at a full charge state of 3.0V for the first cycle.

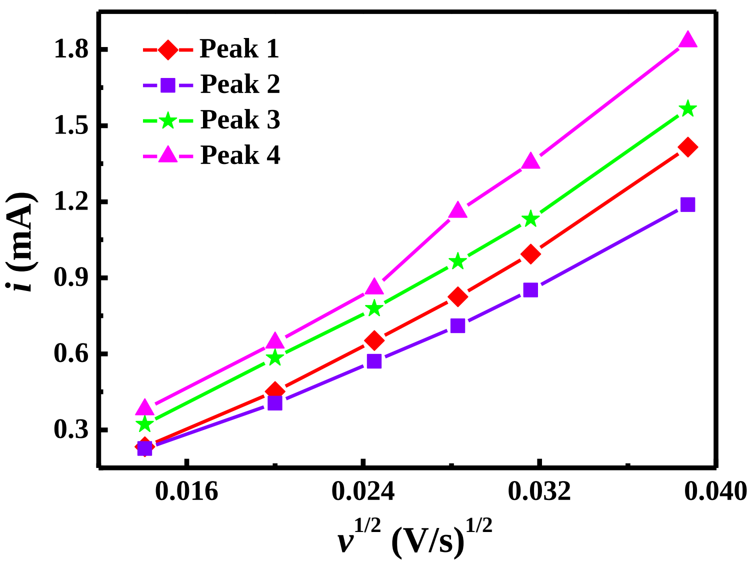

Fig. S12 i vs $v^{1/2}$ plots at each redox peak of CV profiles (peak current: i, scan rate: v).

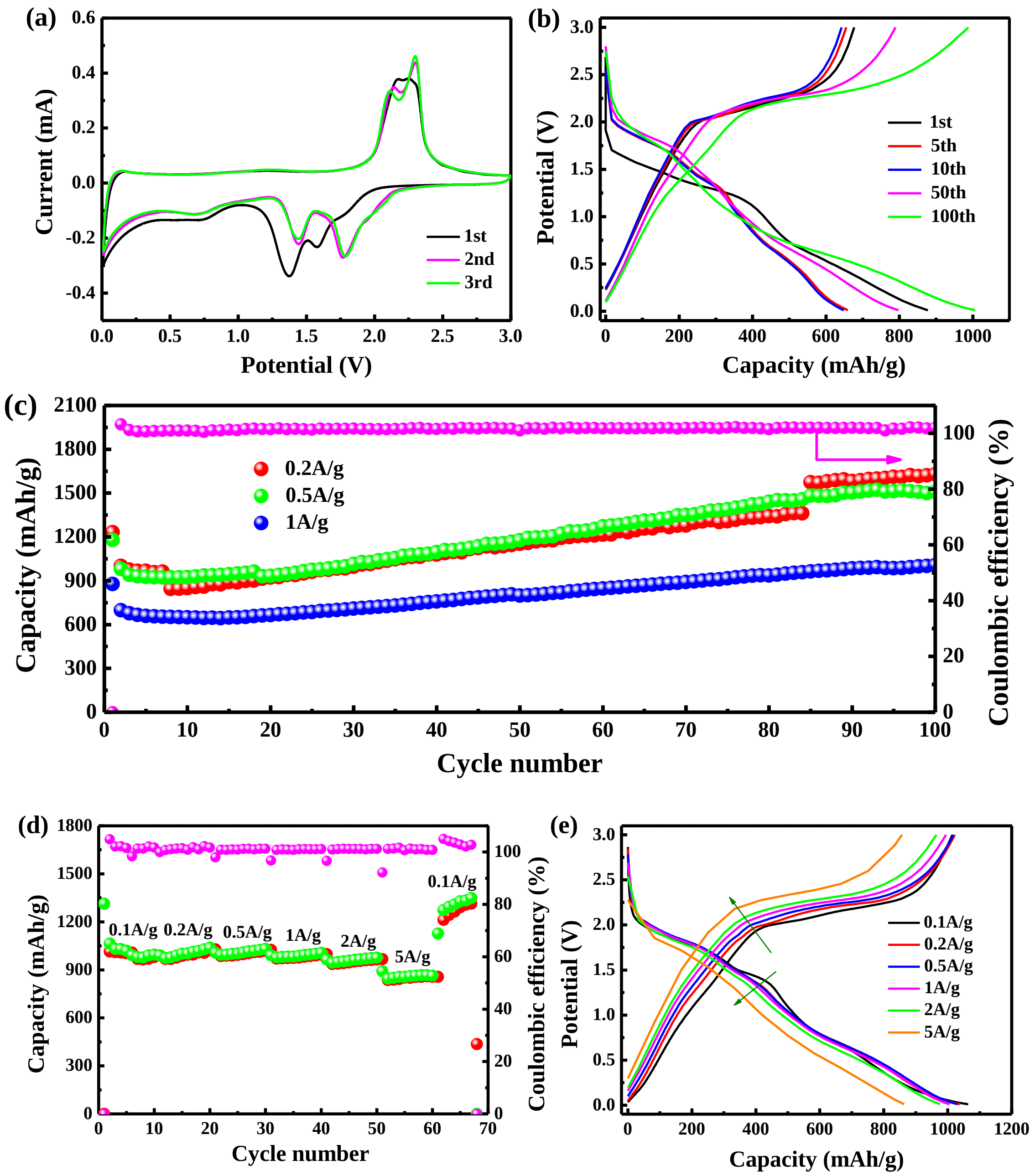

Fig. S13 Electrochemical performances of $CoSe_2$@N-CF/CNTs in the voltage range of 3.0-0.01 V vs Li/Li^+: (a) CV curves at a scan rate of 0.2 $mV \cdot s^{-1}$. (b) Voltage-capacity curves at a current density of 1 A/g. (c) Comparison of cycling performance at the current rates of 0.2, 0.5 and 1 A/g. (d) Rate capability at increasing current density from 0.1 A/g to 5 A/g. (e) The corresponding galvanostatic charge/discharge profiles at various rates.

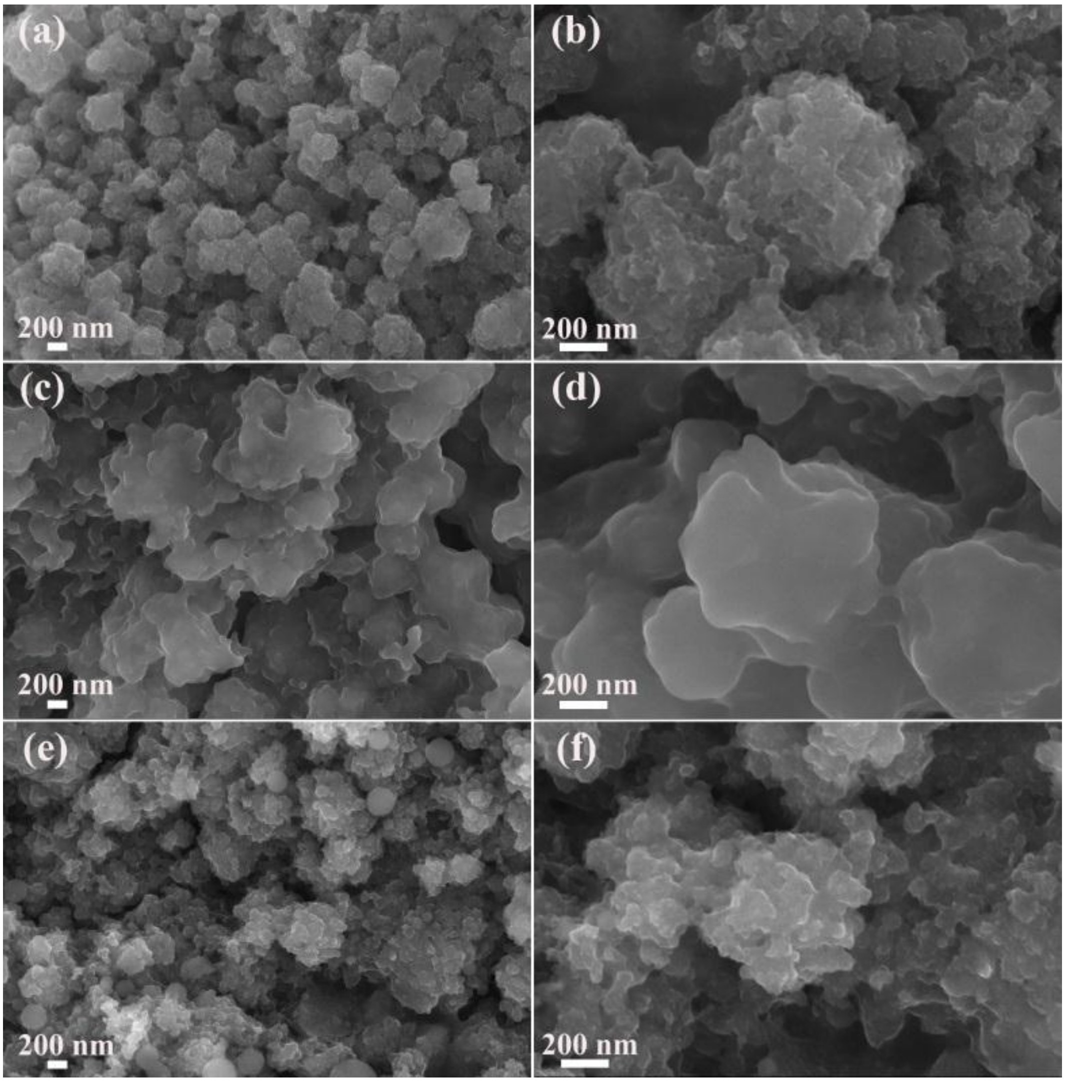

Fig. S14 Representative SEM images of the $CoSe_2$@N-CF/CNTs electrode after 100 cycles at 1A g^{-1}: (a, b) in the voltage range of 0.5-3.0 V for LIBs; (c, d) in the voltage range of 0.01-3.0 V for LIBs and (e, f) for SIBs.

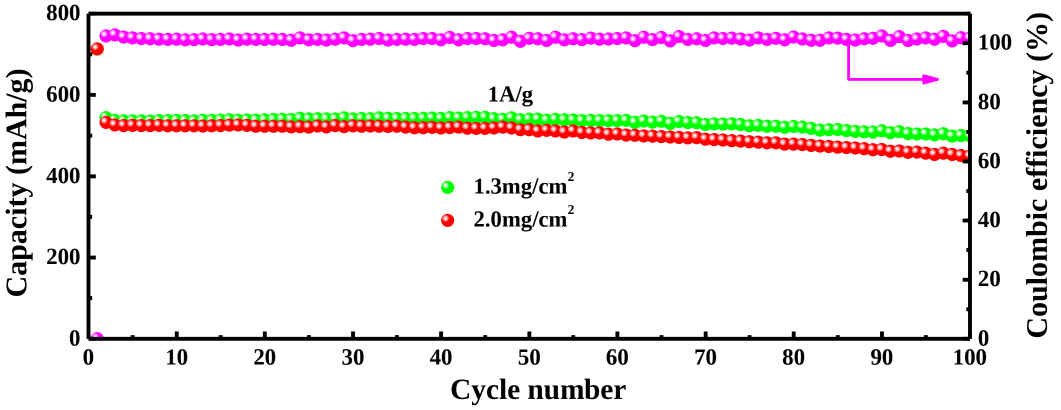

Fig. S15 Cycling performances of the $CoSe_2$@N-CF/CNTs electrode in SIBs at 1.3 and 2.0 mg·cm^{-2} $CoSe_2$ loadings.

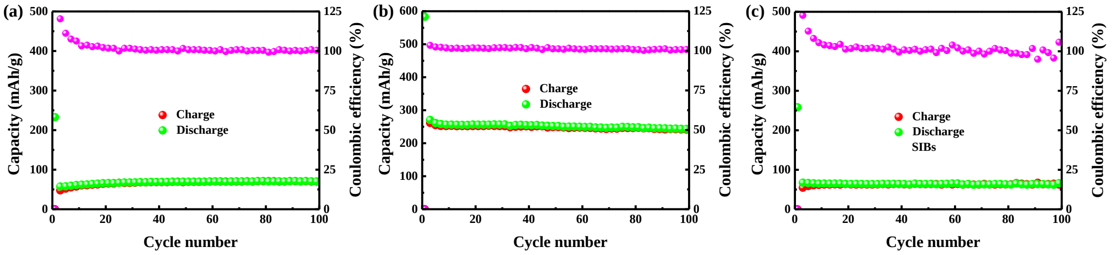

Fig. S16 Cycle performance of the carbon matrix at 1 $A \cdot g^{-1}$ for LIBs in the voltage range of (a) 0.5-3.0 V and (b) 0.01-3.0 V, and (c) for SIBs.

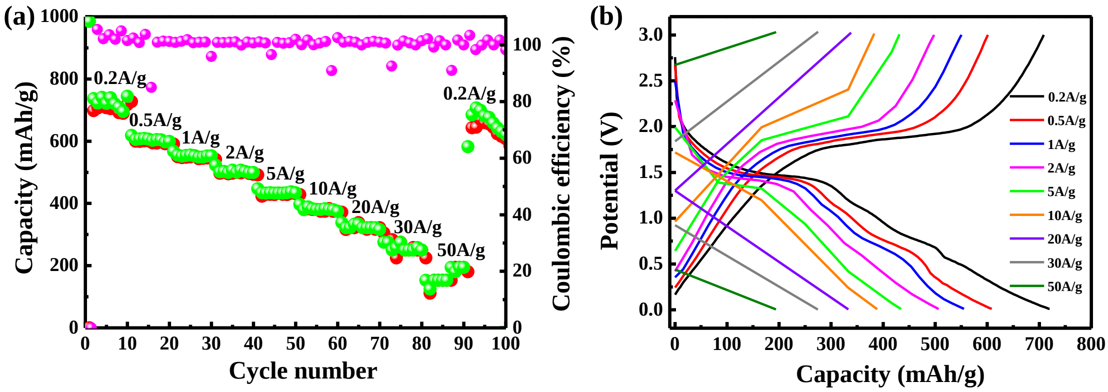

Fig. S17 (a) Rate performance of the $CoSe_2$@N-CF/CNTs for SIBs at increasing current density from 0.2 $A \cdot g^{-1}$ to 50 $A \cdot g^{-1}$. (b) The corresponding charge/discharge profiles at various rates.

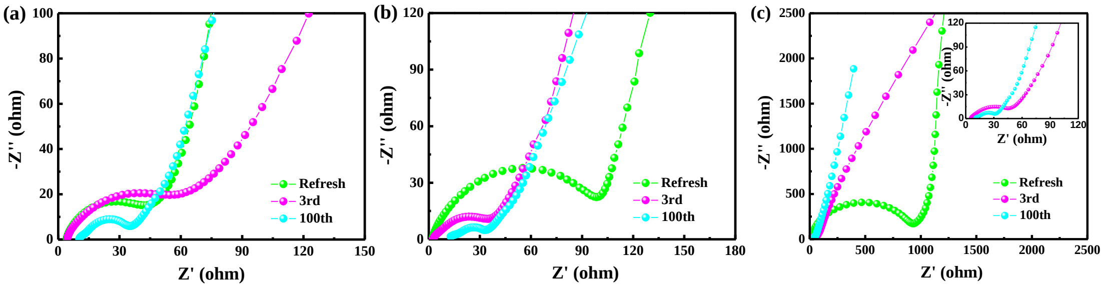

Fig. S18 Nyquist plots of $CoSe_2$@N-CF/CNTs in the cut-off voltage range of (a) 0.5-3.0 V and (b) 0.01-3.0 V for LIBs and (c) for SIBs at different cycles in the frequency range of 100 kHz-100mHz. The inset in panel (c) is the zoom-in image of panel (c) in the range of 0-120 ohm.

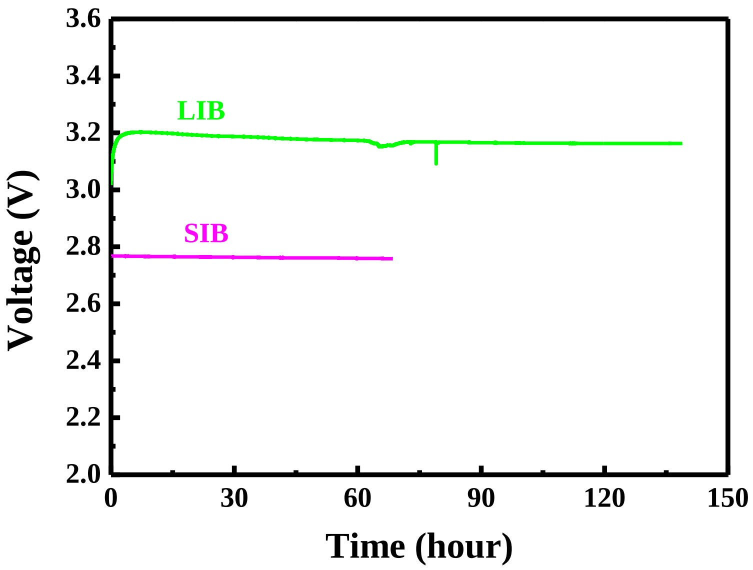

Fig. S19 Changes in open circuit voltage (OCV) with measured time.

Table S1. Specific surface area, pore size and pore volume of the prepared composites

Samples	Specific surface area (m^2/g)	Total pore volume (cm^3/g)	Average pore size (nm)
Co@N-CF/CNTs	277.59	0.584	8.41
Co@CF/CNTs	237.96	0.398	6.68
$CoSe_2$@N-CF/CNTs	17.09	0.134	31.39
$CoSe_2$@CF/CNTs	43.33	0.146	13.44
$CoSe_2$@N-CF/CNTs-3h	20.34	0.096	18.88
$CoSe_2$@N-CF/CNTs-6h	18.58	0.091	19.60
$CoSe_2$@N-CF/CNTs-24h	19.33	0.090	18.70

Table S2. Element contents (atomic percentage) for Co@N-CF/CNTs and Co@CF/CNTs obtained from XPS analysis.

Samples	C	N	O	Co
Co@N-CF/CNTs	80.64%	7.53%	8.76%	3.07%
Co@CF/CNTs	89.03%	1.01%	7.79%	2.17%

Table S3. Summary of structural parameters derived from XRD measurements.

Samples	lattice plane	β (rad)	2θ (°)	$d = 0.89\lambda/\beta\cos\theta$ (nm)
$CoSe_2$@N-CF/CNTs	(111)	0.251	34.38	32.77
$CoSe_2$@CF/CNTs	(111)	0.372	34.48	22.12
$CoSe_2$@N-CF/CNTs-3h	(111)	0.6	34.38	13.71
$CoSe_2$@N-CF/CNTs-6h	(111)	0.593	34.39	13.87
$CoSe_2$@N-CF/CNTs-24h	(111)	0.597	34.37	13.78

Table S4. A survey of electrochemical properties of various typical metal chalcogenides as anode materials for LIBs reported in recent literatures.

Material	Mass loading (mg/cm^2)	Voltage range (V vs Li/Li^{+1})	Current density (A/g)	Cycle number	Capacity (mAh/g)	Ref
CoS_x	1.0–1.5	0.01–3.0	0.5	100	1012.1	[1]
CoS<br>CoS_2	/	0.5–3.0	0.1<br>0.1	10<br>10	589<br>720	[2]
M-CoS@C	1.0	0.01–3.0	1.0	500	790	[3]
CoS@PCP/CNTs	/	0.01–3.0	0.2	100	1668	[4]
N-doped carbon@CoS	/	0–3.0	1.0<br>2.0	150<br>1400	1150<br>671	[5]
CoS_2@NG	/	0.01–3.0	0.1	150	882	[6]
NC/CoS_2-650	/	0.1–3.0	0.1	50	560	[7]
Co_3S_4-PNS/GS	1.0–1.5	0.005–3.0	0.5	200	672	[8]
Co_9S_8	1.5–2.0	1.0–3.0<br>0.01–3.0	0.5<br>1.0	150<br>350	365<br>1300	[9]
Co_9S_8-650@C	1.0	0.01–3.0	0.1<br>2.0	100<br>800	1414<br>896	[10]
Bi_2Se_3	/	0.01–3.0	0.05	50	45	[11]
WSe_2	1.0	0.01–3.0	0.1C	30	530	[12]
NiSe/C	/	0.01–3.0	0.1	50	428	[13]
α-MnSe	/	0–3.0	2C	5000	150	[14]
α-FeSe@C	1.5–2.0	1.0–3.0	0.04	40	340	[15]
VSe_2/graphene	/	0.01–3.0	0.1	60	630	[16]
Sb_2Se_3	/	0.3–2.5	5μA/cm^{-2}	100	530.5	[17]
Sb_2Se_3 nanosheets	/	0.01–3.0	0.05	30	298	[18]
ZnSe/C	3.0	0–3.0<br>0–2.0	0.1<br>0.1	300<br>300	705<br>437	[19]
ZnSe-rGO	1.0	0.01–3.0	0.1<br>1.0	50<br>400	876<br>778	[20]
$MoSe_2$	/	0.1–3.0	0.1C	50	405	[21]
$MoSe_2$/rGO	/	0.01–3.0	0.1C<br>0.5C	50<br>600	650<br>470	[22]

$MoSe_2$@PHCS	0.9–1.1	0.01–3.0	1	100	681	[23]
SnSe	2–3	0.01–3.0	0.2	80	676	[24]
SnSe/C	0.8–1.2	0.01–3.0	0.5	100	633.1	[25]
SnSe@CNFs	/	0.01–2.5	0.2	100	840	[26]
$SnSe_2$/RGO	0.95	0.001–3.0	0.04	30	640	[27]
$Co_{0.85}Se$	2.5–3.5	0.01–3.0	0.2<br>0.5	50<br>50	516<br>295	[28]
rGO/$CoSe_2$	/	0.01–3.0	0.2	200	1577	[29]
CoSe@PCP	1.8	0.005–3.0	0.2<br>1.0	100<br>500	675<br>708.2	[30]
CoSe@carbon-nanoboxes	1.0–1.4	0.01–3.0	0.2<br>0.5<br>1.0	100<br>100<br>100	860<br>711<br>660	[31]
$CoSe_2$@CNFs	/	0.01–3.0	0.2	300	1405	[32]
This work	1.5–2.0	0.5–3.0	1.0	500	428	
		0.01–3.0	0.2<br>0.5<br>1.0	100<br>100<br>100	1629<br>1508<br>1006	

Table S5. A survey of electrochemical properties of various typical metal chalcogenides as anode materials for SIBs reported in the previous literatures

Material	Electrolyte	Mass loading (mg/cm^2)	Voltage range (V)	Current density (A/g)	Cycle number	Capacity (mAh/g)
Co_3S_4-PNS/GS (Ref.[8])	$NaClO_4$/EC/DEC/FEC	1.0–1.5	0.005–3.0	0.5	50	329
SnSe/C (Ref.[25])	$NaClO_4$/EC/DEC/FEC	0.8–1.2	0.01–2.0	0.5	200	324.9
CoS_x (Ref.[1])	$NaClO_4$/EC/PC/FEC	1.0–1.5	0.01–3.0	0.5	100	572
CoSe@PCP (Ref.[30])	$NaClO_4$/EC/PC/FEC	1.8	0.005–3.0	0.1	100	341
N-doped carbon@CoS (Ref.[5])	$NaClO_4$/EC/DEC	/	0–3.0	3.0	1400	220
$SnSe_2$/RGO (Ref.[33])	$NaClO_4$/EC/DMC	1.2	0.005–2.5	0.1	100	515
Cu_2Se (Ref.[34])	$NaClO_4$/EC/DMC	0.66	1.8–2.5	0.025	100	113.6
CoS@rGO (Ref.[35])	$NaCF_3SO_3$/DEGDME	1.0	0.6–2.9	1.0	1000	420
SnSSe (Ref.[36])	$NaCF_3SO_3$/DEGDME	1.75	0.1–3.0<br>0.5–3.0	5.0<br>0.5	1000<br>600	330<br>400
Urchin-like $CoSe_2$ (Ref.[37])	$NaCF_3SO_3$/DEGDME	1.3	0.5–3.0	1.0	1800	410
$CoSe_2$@C/CNTs (Ref.[38])	$NaCF_3SO_3$/DEGDME	0.7–0.8	0.5–2.9	1.0	1000	390
M-CoS@C (Ref.[3])	$NaClO_4$/PC/FEC	1.0	0.01–3.0	0.2	100	532
CoSe/C (Ref.[39])	$NaClO_4$/PC/FEC	1.0	0.01–3.0	0.5	50	531.6
CoSe⊂carbon NWs (Ref.[40])	$NaClO_4$/PC/FEC	/	0.005–3.0	0.2<br>0.2	100<br>200	299<br>266
WSe_2/C (Ref.[41])	$NaClO_4$/EC/DMC/FEC	1.0–1.2	0.01–3.0	0.2	50	270
$MoSe_2$@PHCS (Ref.[23])	$NaClO_4$/EC/DMC/FEC	0.9–1.1	0.01–3.0	0.2	100	580
$MoSe_2$ microspheres (Ref.[42])	$NaClO_4$/EC/DMC/FEC	/	0.001–3.0	0.2	50	433
$FeSe_2$-AC	$NaClO_4$/EC/DMC/FEC	1.2	0.001–3.0	0.5	150	379

(Ref.[43])						
NiSe/C<br>(Ref.[13])	$NaClO_4$/EC/DMC/FEC	/	0.01–3.0	0.1	50	280
$NiSe_2$-rGO-C<br>(Ref.[44])	$NaClO_4$/EC/DMC/FEC	1.2	0.001–3.0	0.2	100	468
$CoSe_2$@N-PGC/CNTs<br>(Ref.[45])	$NaClO_4$/EC/DMC/FEC	1.2	0.001–3.0	0.2<br>1.0	100<br>400	420<br>300.2
Co_9Se_8/rGO<br>(Ref.[46])	$NaPF_6$/EC/DMC/FEC	1.0	0.01–3.0	0.05	100	406
$CoSe_2$-GC<br>(Ref.[47])	$NaClO_4$/EC/DMC/FEC	1.2	0.001–3.0	0.2	100	393
$CoSe_x$-rGO<br>(Ref.[48])	$NaClO_4$/EC/DMC/FEC	1.2	0.001–3.0	0.3	50	420
$CoSe_2$ microspheres<br>(Ref.[49])	$NaClO_4$/EC/DMC/FEC	/	0.001–3.0	0.5	40	467
This work	$NaClO_4$/EC/DMC/FEC	1.5–2.0	0.01–3.0	0.1<br>0.5<br>1.0<br>2.0	100<br>100<br>100<br>100	606<br>523<br>499<br>450

References

[1] Y. Xiao, J. Y. Hwang, I. Belharouak, Y. K. Sun, Nano Energy 2017, 32, 320.

[2] Y. Wang, J. J. Wu, Y. F. Tang, X. J. Lii, C. Y. Yang, M. S. Qin, F. Q. Huang, X. Li, X. Zhang, ACS Appl Mater Inter 2012, 4, 4246.

[3] Q. D. Li, L. Li, K. A. Owusu, W. Luo, Q. Y. An, Q. L. Wei, Q. J. Zhang, L. Q. Mai, Nano Energy 2017, 41, 109.

[4] R. B. Wu, D. P. Wang, X. H. Rui, B. Liu, K. Zhou, A. W. K. Law, Q. Y. Yan, J. Wei, Z. Chen, Adv Mater 2015, 27, 3038.

[5] Y. M. Chen, X. Y. Li, K. Park, L. M. Zhou, H. T. Huang, Y. W. Mai, J. B. Goodenough, Angew Chem Int Edit 2016, 55, 15831.

[6] W. D. Qiu, J. Q. Jiao, J. Xia, H. M. Zhong, L. P. Chen, Chem-Eur J 2015, 21, 4359.

[7] Q. F. Wang, R. Q. Zou, W. Xia, J. Ma, B. Qiu, A. Mahmood, R. Zhao, Y. Y. C. Yang, D. G. Xia, Q. Xu, Small 2015, 11, 2511.

[8] Y. C. Du, X. S. Zhu, X. S. Zhou, L. Y. Hu, Z. H. Dai, J. C. Bao, J Mater Chem A 2015, 3, 6787.

[9] J. Liu, C. Wu, D. D. Xiao, P. Kopold, L. Gu, P. A. van Aken, J. Maier, Y. Yu, Small 2016, 12, 2354.

[10] Y. L. Zhou, D. Yan, H. Y. Xu, J. K. Feng, X. L. Jiang, J. Yue, J. Yang, Y. T. Qian, Nano Energy 2015, 12, 528.

[11] H. M. Xu, G. Chen, R. C. Jin, D. H. Chen, Y. Wang, J. Pei, RSC Adv 2014, 4, 8922.

[12] F. J. Chen, J. Wang, B. Li, C. H. Yao, H. F. Bao, Y. F. Shi, Mater Lett 2014, 136, 191.

[13] Z. A. Zhang, X. D. Shi, X. Yang, Electrochim Acta 2016, 208, 238.

[14] N. Li, Y. Zhang, H. Y. Zhao, Z. Q. Liu, X. Y. Zhang, Y. P. Du, Inorg Chem 2016, 55, 2765.

[15] D. H. Wei, J. W. Liang, Y. C. Zhu, L. Hu, K. L. Zhang, J. J. Zhang, Z. Q. Yuan, Y. T. Qian, Electrochem Commun 2014, 38, 124.

[16] Y. P. Wang, B. B. Qian, H. H. Li, L. Liu, L. Chen, H. B. Jiang, Mater Lett 2015, 141, 35.

[17] M. Z. Xue, Z. W. Fu, J Alloy Compd 2008, 458, 351.

[18] R. C. Jin, Z. Q. Liu, L. X. Yang, J. S. Liu, Y. B. Xu, G. H. Li, J Alloy Compd 2013, 579, 209.

[19] H. T. Kwon, C. M. Park, J Power Sources 2014, 251, 319.

[20] Z. Zhang, Y. Fu, X. Yang, Y. H. Qu, Q. Li, Electrochim Acta 2015, 168, 285.

[21] H. Wang, X. Y. Wang, L. Wang, J. Wang, D. L. Jiang, G. P. Li, Y. Zhang, H. H. Zhong, Y. Jiang, J Phys Chem C 2015, 119, 10197.

[22] J. Y. Yao, B. R. Liu, S. Ozden, J. J. Wu, S. B. Yang, M. T. F. Rodrigues, K. Kalaga, P. Dong, P. Xiao, Y. H. Zhang, R. Vajtai, P. M. Ajayan, Electrochim Acta 2015, 176, 103.

[23] X. Yang, Z. A. Zhang, Y. Fu, Q. Li, Nanoscale 2015, 7, 10198.

[24] X. F. Wang, B. Liu, Q. Y. Xiang, Q. F. Wang, X. J. Hou, D. Chen, G. Z. Shen, Chemsuschem 2014, 7, 308.

[25] Z. A. Zhang, X. X. Zhao, J. Li, Electrochim Acta 2015, 176, 1296.

[26] L. Zhang, L. Lu, D. C. Zhang, W. T. Hu, N. Wang, B. Xu, Y. M. Li, H. Zeng, Electrochim Acta 2016, 209, 423.

[27] J. Choi, J. Jin, I. G. Jung, J. M. Kim, H. J. Kim, S. U. Son, Chem Commun 2011, 47, 5241.

[28] J. S. Zhou, Y. Wang, J. Zhang, T. Chen, H. H. Song, H. Y. Yang, Nanoscale 2016, 8, 14992.

[29] Z. P. Li, H. T. Xue, J. Q. Wang, Y. B. Tang, C. S. Lee, S. R. Yang, Chemelectrochem 2015, 2, 1682.

[30] J. B. Li, D. Yan, T. Lu, Y. F. Yao, L. K. Pan, Chem Eng J 2017, 325, 14.

[31] H. Hu, J. T. Zhang, B. Y. Guan, X. W. Lou, Angew Chem Int Edit 2016, 55, 9514.

[32] J. K. Wang, H. K. Wang, D. X. Cao, X. Lu, X. G. Han, C. M. Niu, Part Part Syst Char 2017, 34.

[33] F. Zhang, C. Xia, J. J. Zhu, B. Ahmed, H. F. Liang, D. B. Velusamy, U. Schwingenschlogl, H. N. Alshareef, Adv Energy Mater 2016, 6.

[34] J. L. Yue, Q. Sun, Z. W. Fu, Chem Commun 2013, 49, 5868.

[35] S. J. Peng, X. P. Han, L. L. Li, Z. Q. Zhu, F. Y. Cheng, M. Srinivansan, S. Adams, S. Ramakrishna, Small 2016, 12, 1359.

[36] X. S. Wang, D. Chen, Z. H. Yang, X. H. Zhang, C. Wang, J. T. Chen, X. X. Zhang, M. Q. Xue, Adv Mater 2016, 28, 8645.

[37] K. Zhang, M. H. Park, L. M. Zhou, G. H. Lee, W. J. Li, Y. M. Kang, J. Chen, Adv Funct Mater 2016, 26, 6728.

[38] Y. C. Tang, Z. B. Zhao, X. J. Hao, Y. W. Wang, Y. Liu, Y. N. Hou, Q. Yang, X. Z. Wang, J. S. Qiu, J Mater Chem A 2017, 5, 13591.

[39] Y. F. Zhang, A. Q. Pan, L. Ding, Z. L. Zhou, Y. P. Wang, S. Y. Niu, S. Q. Liang, G. Z. Cao, ACS Appl Mater Inter 2017, 9, 3624.

[40] C. Wu, Y. Jiang, P. Kopold, P. A. van Aken, J. Maier, Y. Yu, Adv Mater 2016, 28, 7276.

[41] Z. A. Zhang, X. Yang, Y. Fu, RSC Adv 2016, 6, 12726.

[42] Y. N. Ko, S. H. Choi, S. B. Park, Y. C. Kang, Nanoscale 2014, 6, 10511.

[43] G. D. Park, J. H. Kim, Y. C. Kang, Mater Charact 2016, 120, 349.

[44] J. S. Cho, S. Y. Lee, Y. C. Kang, Sci Rep-Uk 2016, 6.

[45] S. K. Park, J. K. Kim, Y. C. Kang, Chem Eng J 2017, 328, 546.

[46] X. F. Wang, D. Z. Kong, Z. X. Huang, Y. Wang, H. Y. Yang, Small 2017, 13.

[47] J. S. Cho, J. M. Won, J. K. Lee, Y. C. Kang, Nano Energy 2016, 26, 466.

[48] G. D. Park, Y. C. Kang, Chem-Eur J 2016, 22, 4140.

[49] Y. N. Ko, S. H. Choi, Y. C. Kang, ACS Appl Mater Inter 2016, 8, 6449.
